# Deeply subwavelength phonon-polaritonic crystal made of a van der Waals material

**DOI:** 10.1038/s41467-018-07795-6

**Published:** 2019-01-03

**Authors:** F. J. Alfaro-Mozaz, S. G. Rodrigo, P. Alonso-González, S. Vélez, I. Dolado, F. Casanova, L. E. Hueso, L. Martín-Moreno, R. Hillenbrand, A. Y. Nikitin

**Affiliations:** 1 CIC nanoGUNE, 20018 Donostia-San Sebastián, Spain; 2 Instituto de Ciencia de Materiales de Aragón and Departamento de Física de la Materia Condensada, CSIC-Universidad de Zaragoza, 50009 Zaragoza, Spain; 3 Centro Universitario de la Defensa, Ctra. de Huesca s/n, 50090 Zaragoza, Spain; 4 Departamento de Física, Universidad de Oviedo, 33007 Oviedo, Spain; 5 IKERBASQUE, Basque Foundation for Science, 48013 Bilbao, Spain; 6 CIC nanoGUNE and UPV/EHU, 20018 Donostia-San Sebastián, Spain; 7 Donostia International Physics Center (DIPC), 20018 Donostia-San Sebastián, Spain; 8 Present Address: Department of Materials, ETH Zürich, 8093 Zürich, Switzerland

## Abstract

Photonic crystals (PCs) are periodically patterned dielectrics providing opportunities to shape and slow down the light for processing of optical signals, lasing and spontaneous emission control. Unit cells of conventional PCs are comparable to the wavelength of light and are not suitable for subwavelength scale applications. We engineer a nanoscale hole array in a van der Waals material (h-BN) supporting ultra-confined phonon polaritons (PhPs)—atomic lattice vibrations coupled to electromagnetic fields. Such a hole array represents a polaritonic crystal for mid-infrared frequencies having a unit cell volume of ${\mathrm{10}}^{{\mathrm{ - 5}}}{\lambda}_{\mathrm{0}}^{\mathrm{3}}$ (with *λ*_0_ being the free-space wavelength), where PhPs form ultra-confined Bloch modes with a remarkably flat dispersion band. The latter leads to both angle- and polarization-independent sharp Bragg resonances, as verified by far-field spectroscopy and near-field optical microscopy. Our findings could lead to novel miniaturized angle- and polarization-independent infrared narrow-band couplers, absorbers and thermal emitters based on van der Waals materials and other thin polar materials.

## Abstract (web summary)

Photonic crystals can steer, shape, and sculpture the flow of photons. Here, the author fabricate a deep-subwavelength photonic crystal slab that supports ultra-confined phonon polaritons, by patterning a nanoscale hole array in h-BN.

## Introduction

Photonic crystals (PCs) offer the possibility to manipulate the flow of light, to enhance light–matter interactions in numerous opto-electronic technologies and quantum optical devices, and to control the spontaneous emission rate of local emitters^1,2^. At mid-infrared (IR) frequencies, PCs find applications as thermal emitters, optical couplers^3^, devices for chemical and biological spectroscopy^4^, and sensors for environmental monitoring (e.g., gas sensing)^5^. They are normally fabricated by either patterning Si slabs^6^ or metal layers^7^ that are combined with quantum wells operating at the desired wavelength. In both cases the value of the refractive index of the supported electromagnetic modes, *n*, is rather low (e.g., *n* ~ 3 for Si), thus restricting the confinement of light and putting limitations to the dimension of PCs and potential PC-based integrated circuits (for example, room-temperature IR subwavelength photodetectors^8,9^). Low *n* also implies a steep dispersion of the PC modes (close to the light cone), leading to a broadening of the resonances in IR PCs and an angle-dependent absorption/emission^7^. The light confinement can be improved by means of polaritons, which are modes formed by the coupling of dipolar excitations and electromagnetic fields^10^. Structured thin films of doped semiconductors^11,12^ and graphene^13,14^ supporting plasmon polaritons, or polar dielectrics (such as SiO_2_, Al_2_O_3_ or SiC^15–19^) supporting phonon polaritons (PhPs), can be seen as “polaritonic crystals”^20^—lattices with periods comparable to the polariton wavelengths. However, the intrinsic losses of these materials are relatively high (the quality factor *Q* of the resonances are limited to 30), with the exception of SiC slabs (where PhPs have long lifetimes)^17–19^, whose fabrication presents substantial practical difficulties.

Promising alternative materials for IR polaritonic crystals can be found among many low-dimensional van der Waals (vdW) crystals that support a diversity of polaritons with unique properties (enormous confinement, tunability, low losses, or negative phase velocity, among others). They are thus of large interest for the growing field of nanophotonics^21,22^. Particularly, in the mid-infrared frequency range, hexagonal boron nitride (h-BN) crystals exhibit anisotropic phonons and support Type I (*ε*_*z*_ < 0, *ε*_*x*,*y*_ > 0) and Type II (*ε*_*z*_ > 0, *ε*_*x*,*y*_ < 0) hyperbolic phonon polaritons (HPhPs) inside the lower and upper Reststrahlen bands, respectively^23,24^. In h-BN slabs, HPhPs propagate in the form of waveguide modes M*n*, with *n* = 0, 1, 2, …,^23–26^ with both their wavelength and propagation length decreasing with *n*. Due to their remarkably long lifetimes^25,27^, HPhPs in h-BN can be used for molecular vibration spectroscopy and strong coupling^28^. Moreover, the preparation of thin h-BN slabs is a well-established process, as well as its structuring into cones^24^, rods^29^ and stripes^28^, in which HPhPs exhibit sharp Fabry–Perot (“cavity”) resonances. Despite these promising initial works, periodically structured h-BN films in which HPhPs can exhibit “collective” modes have not been considered yet, so that until now the concept of a h-BN PhP crystal has remained unexplored (although h-BN-based “conventional” PCs have been demonstrated at the visible frequencies, where HPhPs are not supported^30^).

Here we propose, design and fabricate a mid-infrared polaritonic crystal formed by a rectangular hole array (HA) in a thin h-BN flake. We tune the polaritonic crystal period to match the wavelengths of the M0 HPhP mode (with the longest propagation length) in the upper Reststrahlen band. By mid-infrared spectroscopy we reveal narrow, geometrically tunable Bragg resonances. We associate them with an extremely flat polaritonic band formed by highly confined (deeply subwavelength) Bloch modes, which we verify by near-field optical microscopy. Due to the strong confinement of the modes, the resonances are intrinsically independent of the angle and polarization (due to the symmetry of the HA) of the illumination, while the whole size of the polaritonic crystal becomes comparable to a single free space wavelength. The idea of a vdW polaritonic crystals thus goes far beyond the original concept by Yablonovitch^31^ and enables the generalization of all the PC-based photonics to a deeply subwavelength scale. The vdW polaritonic crystal can be also seen as a counterpart of vdW hyperbolic metasurfaces^32^, in which the period of the structuring of h-BN film is much smaller than the HPhP wavelength.

## Results

### Spectroscopic analysis of the hole arrays

**Fig. 1:**
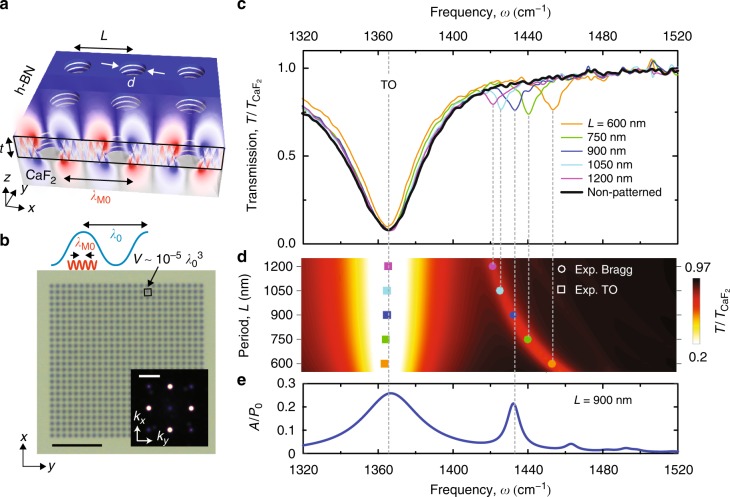
Far-field spectroscopy of the h-BN polaritonic crystal. **a** Schematics of a hole array in a h-BN slab with thickness *t* = 38 nm on a CaF_2_ substrate. **b** Optical image of a hole array with period *L* = 600 nm. The blue and red oscillations represent the wavelength of light in free space, *λ*_0_, and that of the M0 mode, *λ*_M0_, respectively. The inset shows the Fourier transform of the optical image. **c** Experimental normalized transmission spectra for the hole arrays with *L* ranging from 600 to 1200 nm. **d** Simulated transmission as a function of frequency and *L* (colormap). The points indicate the position of the dips from the experimental spectra. **e** Simulated absorption spectra of the hole array with *L* = 900 nm. Scale bar in (**b**): 5 μm. Scale bar in the inset of (**b**): 2π/600 nm^−1^

Figure 1a shows a schematic of the polaritonic crystal considered in this work. It presents a square array of circular holes (diameter *d* = 300 nm), designed to exhibit HPhP resonances at mid-IR frequencies (1360–1480 cm^−1^). The holes are etched in a h-BN slab (with a thickness *t* = 38 nm) on a transparent (CaF_2_) substrate (see Methods). The period of the array, *L*, ranges from 600 to 1200 nm, being thus about one order of magnitude smaller than the illuminating wavelength. It consequently exhibits ultra-small unit cell volumes of about $10^{ - 5}\lambda _0^3$. An optical microscopy image of the resulting structure is shown in Fig. 1b, where the Fourier transform (FT) has prominent sharp peaks at $\vec k = ( \pm G,0)$ and $\vec{k} = (0, \pm G)$ with *G* = 2*π*/*L*, as expected for a square lattice.

To optically characterize the polaritonic crystals, we performed Fourier transform infrared spectroscopy (FTIR) experiments using unpolarized thermal radiation at normal incidence. The resulting transmission spectra, $T{/}T_{\mathrm{CaF}_2}$, are shown in Fig. 1c (color curves) together with the spectrum obtained for a bare h-BN slab (black curve). Apart of a strong dip corresponding to the h-BN transverse optical (TO) phonon (*ω*_TO_ = 1366 cm^−1^), the spectra of the polaritonic crystals show a sharp dip (with an estimated *Q*-factor of 190) at larger frequencies with a spectral position depending on *L*. To better analyze this result, the spectral positions of both the TO phonon dip (squares) and period-dependent dip (circles) are plotted in Fig. 1d as a function of both *L* and *ω*, together with the numerically simulated transmission spectra $T{/}T_{\mathrm{CaF}_2}$. In both experiment and theory, we observe that the frequency of the second dip strongly decreases with increasing *L*, while the minima of the h-BN TO dip is not affected by the periodic structuring. Remarkably, according to the simulations, the value of the absorption of the second peak (Fig. 1e) is comparable to that of the TO peak, revealing the high coupling efficiency provided by the HA. These results demonstrate that the transmission dips in the spectra of our deeply subwavelength polaritonic crystals can be tuned by *L*, in a similar fashion to the “geometrical” plasmonic resonances in metallic hole arrays at visible frequencies^20^, the latter, however, having significantly lower *Q*-factors (by a factor of 10).

To understand the origin of the period-dependent transmission dips in our polaritonic crystals, we simulated the field distribution, Re[*E*_*z*_(*x*, *z*)] (Fig. 1a), at the wavelength of the dip minimum for *L* = 900 nm. A “zigzag” ray pattern inside the slab (*x–z* plane) is observed, evidencing the excitation of many HPhP modes with different wavelengths at the edges of the holes^25,33,34^. However, the field distribution outside of the slab is very different, as we see field oscillations of a single periodicity (alternating red and blue lobes). The period of the oscillations matches with the wavelength of the HPhP M0 mode (found from the mode dispersion^23^), and the nodes are located at the center of the holes. The field distribution thus resembles that of a standing wave, which permits us to identify the period-dependent dip in the polaritonic crystal transmission spectra as the first-order Bragg resonance of a HPhP M0 mode. Importantly, the analysis of the propagation length, *l*_M0_, of the HPhP M0 mode in the continuous h-BN slab (Supplementary Note 7) reveals that *l*_M0_ > *L* at the frequencies of the transmission dips in all the HAs, which proves that this prerequisite for the formation of the Bragg resonances is fulfilled.

### Near-field imaging of the Bloch mode

**Fig. 2:**
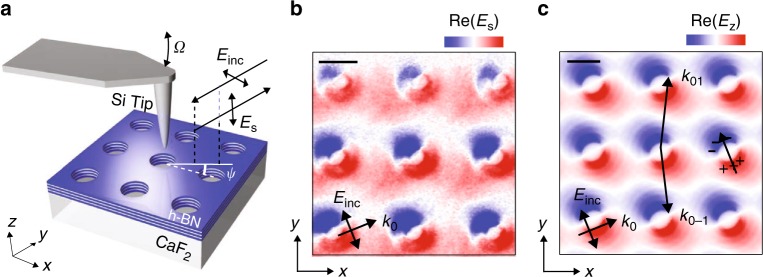
Near-field imaging of a Bloch mode in the h-BN polaritonic crystal. **a** Schematics of the experiment. **b**, **c** Experimental near-field image (**b**) and simulated field distribution (**c**) of the hole array with *L* = 900 nm at the resonant frequency 1428 cm^−1^. Black arrows indicate the direction of the electric field, *E*_inc_, and in-plane wave vector of the incident wave, *k*_0_. The effective induced dipole and the *k*-vectors of (0, 1) and (0, −1) diffraction orders constituting the Bloch mode are indicated in (**c**) by the black arrows. Scale bars in (**b**, **c**): 450 nm

For the experimental verification of the first-order Bragg resonance, we image the field distribution on top of the HA by scattering-type scanning near-field microscopy (s-SNOM), using a weakly scattering Si tip illuminated by obliquely incident s-polarized light (see schematics in Fig. 2a, and details in the Methods) as a probe. Interferometric recording of the scattered p-polarized radiation allows for mapping the vertical electric field of the mode that is excited by the incident wave^35^. The near-field image (showing the real part of the signal, *E*_s_) obtained at the frequency of the dip (1428 cm^−1^, dark blue curve in Fig. 1c) is shown in Fig. 2b. We see field oscillations (red and blue colors indicate the field polarity) that match the period *L* of the polaritonic crystal, thus revealing the spatial field structure of the HPhP M0 Bloch mode in the *y*-direction. The slight rotation of the observed field pattern is attributed to the illumination used in our experiment (the plane of incidence is rotated by the angle *ψ* with respect to one of the HA’s translation vectors), which together with the effective electric dipoles induced by the holes also allows for exciting a Bloch mode in the *x*-direction. We corroborate this experimental result by simulating the field distribution, Re[*E*_*z*_(*x*,*y*)] (Fig. 2c), assuming a similar illumination scheme (both angle and polarization) as in the experiment. A perfect matching between experiment and simulation validates our near-field characterization using a Si tip, which permits to identify the deeply subwavelength Bloch mode excited in the first-order Bragg resonance of the polaritonic crystal. Notice that the imaging of the Bloch modes outside of the light cone is not possible with Si tips, as the applied imaging scheme requires the far-field excitation of the Bloch modes. Alternatively, these modes could be accessed via polaritonic interferometry with s-SNOM^36^, which uses metallic tips that allow for the “local” excitation of highly confined modes. However, polariton-interferometric s-SNOM imaging requires the back-reflection of the Bloch mode from edges or other discontinuities in the polaritonic crystal, and thus strongly complicates the interpretation of the near-field images.

### Band structure of the polaritonic crystal

**Fig. 3:**
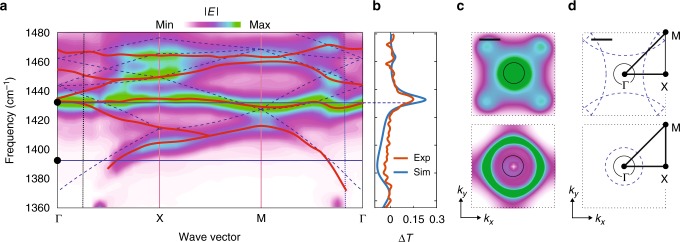
Band structure of hyperbolic phonon polaritons in the h-BN polaritonic crystal. **a** Simulated band structure of the hole array. The colorplot renders the amplitude of the electric field generated by the dipoles, averaged over the unit cell of the array (Methods). The blue dashed lines trace the folded dispersion curve of the HPhP M0 mode in a continuous h-BN slab. The horizontal blue lines mark the frequencies of the plots in (**c**, **d**). **b** Experimental (blue) and calculated (red) normalized difference transmission spectra. **c** Calculated isofrequency plot at 1388.8 cm^−1^ (top) and 1432 cm^−1^ (bottom) for the HA with *L* = 900 nm. **d** Bare isofrequency contours (dashed blue lines). In (**c**, **d**) the black circles represent the light cone, while the dotted black squares represent the first Brillouin zone. Scale bars in (**c**, **d**): 2*k*_0_

To further characterize our deeply subwavelength polaritonic crystal, we perform an analysis of the band structure of the M0 HPhP Bloch modes (see Methods and Supplementary Note 5). Figure 3a shows the calculated band structure of the polaritonic crystal with *L* = 900 nm, along the main directions Γ−*X*−*M*−Γ in the first Brillouin zone. One can recognize features reminiscent of the folded dispersion curves for the M0 HPhP mode in a continuous h-BN slab^23^ (Fig. 3a, the blue dashed lines). At momenta where the folded dispersion curves for the continuous slab intersect (e.g., in the vicinity of the *M*-point at *ω* = 1420 cm^−1^), partial band gaps open, prohibiting the propagation of the M0 mode in the polaritonic crystal. Note that in conventional PCs the band structure is mainly formed in the region of propagating waves, $k \leq \omega {\mathrm{/}}c$. Conversely, the major part of the band structure in our polaritonic crystal is formed outside of the light cone, *k* > *ω*/*c* (Fig. 3a, vertical dashed vertical lines), covering the region of high in-plane momenta modes in the PC, which decay exponentially outside the PC. We complement the band structure analysis by showing in Fig. 3c the FTs of the simulated fields emitted by a vertical point dipole above the HA—counterparts of the isofrequency contours (ICs). The FTs provide information on the density of polaritonic modes in the *k*-space. At low frequencies, the ICs show a circular shape (Fig. 3c, bottom), similar to the bare ICs of the continuous slab, $k_x^2 + k_y^2 = k_{\mathrm{M0}}^2$ (Fig. 3d, bottom), so that at low momenta the density of Bloch polaritonic modes is zero and the light does not couple to the polaritonic crystal (no maxima in the difference transmission signal, Δ*T*, defined as Δ*T* = (–*T* + *T*_bare h-BN_)/*T*_CaF__2_, Fig. 3b). Oppositely, at the frequency of the Bragg resonance, the ICs show a high density of Bloch modes in a large area of the Brillouin zone (Fig. 3c, top), and particularly in the whole area of the light cone (black circle, Fig. 3c, d). The high density of modes is consistent with the band structure of Fig. 3a, where a nearly *k*-independent band for the whole range of momenta is formed at around *ω* = 1430 cm^−1^. Such flat polaritonic bands could be used in ultraslow light applications^37,38^ and for near-field radiative thermal transport^39,40^ at deeply subwavelength scale. Remarkably, the flat band indicates that the coupling to the polaritonic crystal by an incident plane wave can happen for any in-plane momentum (see the maximum in the difference transmission spectra, Δ*T*, Fig. 3b), and thus for any incident angle, *θ* (related to the momentum as *k*_*y*_ = *k*_0_sin*θ*).

### Omnidirectional and polarization-independent absorption peaks

**Fig. 4:**
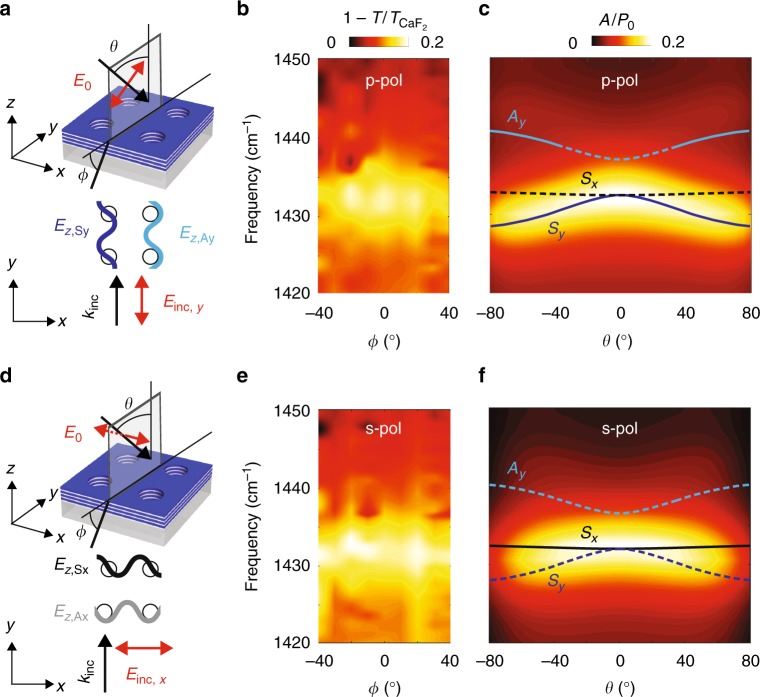
Angle- and polarization-independent HPhP Bragg resonance in the h-BN polaritonic crystal. **a**, **d** Top: schematics of the angle-dependent transmission experiments for p- and s-polarization. Bottom: spatial distribution of the vertical electric field of the Bloch modes in the vicinity of the resonance frequency. **b**, **e** Measured normalized extinction, $1 - T{\mathrm{/}}T_{{\mathrm{CaF}}_2}$, as a function of the stage rotation angle, *ϕ*, and frequency. **c**, **f** Calculated normalized absorption as a function of the incident angle, *θ*, and frequency

We experimentally prove the omnidirectional response of our polaritonic crystal by carrying out optical transmission measurements at oblique incidence for both p- and s-polarizations (see details in Supplementary Notes 5 and 6), schematically shown in Fig. 4a, d. The measured normalized extinction, $1 - T{\mathrm{/}}T_{{\mathrm{CaF}}_2}$, as a function of *θ* and *ω* is represented in Fig. 4b, e. Strikingly, for both polarizations, the extinction maximum is clearly independent upon *θ* in the whole measured range, which is in excellent agreement with the calculated absorption (Fig. 4c, f). A detailed theoretical analysis (based on the perturbation theory and described in the Supplementary Note 5) of both symmetric, *S*, and antisymmetric, *A*, Bloch modes allows us to unambiguously attribute such angle-independent Bragg resonance to the excitation of the Bloch modes *S* (Fig. 4a, d). Namely, the excited modes *S*_*y*_ (p-polarization) and *S*_*x*_ (s-polarization) have an antisymmetric distribution of the vertical electric field with respect to the hole centers, $E_{z,S_y}\sim \sin Gy$ and $E_{z,S_x}\sim \sin Gx$, respectively. This result is also corroborated by the field distribution revealed by our near-field measurements (Fig. 2) and is consistent with previous studies of plasmonic resonances in metallic hole arrays and gratings^2,41,42^. Our findings demonstrate that the narrow Bragg resonances in a h-BN polaritonic crystal are independent of both the illumination angle and polarization. These properties make phonon-polaritonic crystals attractive candidates for narrow-band omnidirectional infrared absorbers, couplers and thermal emitters, significantly smaller than those based on conventional bulk materials^43–45^.

## Discussion

In summary, we have introduced and experimentally realized IR deeply subwavelength polaritonic crystals based on hyperbolic phonon polaritons in nanostructured van der Waals crystal slabs. Such crystals support highly confined Bloch modes with flat bands, giving rise to angle- and polarization-independent geometrically tunable resonances, even in case of the simplest square symmetry. Apart of their potential  application for subwavelength omnidirectional IR absorbers, couplers and reflectors, h-BN polaritonic crystals can be utilized for inhibiting spontaneous emission (the latter, in contrast, being enhanced/accelerated by h-BN optical antennas^24,29,43^). The suppression of spontaneous emission can be achieved by tuning the parameters of the polaritonic crystal (particularly, its symmetry) to open up the full polaritonic bandgap. We note that the layered structure of the van der Waals material (resulting in hyperbolic polaritons) does not play a crucial role for the functionality of the described polaritonic crystal. Similar crystals could be potentially obtained with thin layers of isotropic polar or plasmonic materials, where polaritons have low losses. However, the layered structure of van der Waals materials favors much easier fabrication of high-quality thin layers via exfoliation. Further, PCs based on van der Waals layers could be used as polaritonic hypercrystals (possessing extremely high density of optical states)^44–47^, where several HPhP slab modes are simultaneously explored by superimposing several hole arrays with different periods in the same slab. Furthermore, the combination of h-BN polaritonic crystals/hypercrystals with other low-dimensional materials (such as, e.g., BN-encapsulated graphene^8^) could open the door to integrable hybrid metamaterials with unique opto-electronic properties on the nanoscale.

## Methods

### Fabrication of h-BN hole arrays

For the fabrication of the polaritonic crystals we used h-BN flakes. In order to obtain large and homogeneous h-BN flakes, we first performed mechanical exfoliation of commercially available h-BN crystals (HQ graphene Co, N2A1) using blue Nitto tape (Nitto Denko Co., SPV 224P). Afterwards, the flakes attached to the tape were exfoliated onto several polydimethylsiloxane stamps. The stamps were inspected using an optical microscope and large and homogeneous h-BN flakes were identified and transferred onto a CaF_2_ substrate using the deterministic dry transfer technique^48^.

We patterned the arrays of holes using high-resolution electron beam lithography. The sizes of the arrays were 15 × 15 µm, with a fixed hole diameter (300 nm) and periodicities between 0.6 and 1.2 µm. To that end, we used a single layer polymethyl methacrylate (PMMA) 495 A4 resist as an electron-sensitive resist. The desired holes were written with the electron beam and developed in MIBK:IPA 1:3, resulting in a patterned PMMA layer that is used as a mask to protect the h-BN areas underneath during the etching process. The uncovered h-BN areas were chemically etched in a SF6/Ar 1:1 plasma mixture at 20 sccm flow, 100 mTorr pressure and 100 W power for 60 s (RIE OXFORD PLASMALAB 80 PLUS reactive ion etcher). Finally, the sample was immersed in acetone for several hours for removing the PMMA mask, rinsed in IPA and dried with a N_2_ gun.

To ensure the proper fabrication quality of the hole arrays, we have imaged them by means of both the atomic force microscope (AFM) (performed simultaneously with the optical near-field imaging) and environmental scanning electron microscopy (eSEM) (see the eSEM images in the Supplementary Note 1).

### Far-field spectroscopy measurements

Microspectroscopy transmission spectra of the h-BN arrays were recorded with a Bruker Hyperion 2000 IR microscope coupled to a Bruker Vertex 70 FTIR spectrometer. The IR radiation from a thermal source (Globar) was approximately normal to the surface of the hole array. The spectral resolution was 1 cm^−1^. The area covered by the IR beam was around 10 × 10 μm^2^.

By using a stage that permits to rotate the sample along the *XY* and *XZ* axes, the response of the hole arrays at different incidence angles was studied. The stage rotates the sample from −40° to 40° in the *XZ* axis. It allowed to record transmission spectra with any linear polarization.

### Near-field imaging

Our commercially available s-SNOM (Neaspec, Munich) is based on an AFM. Conventional silicon tips acted as scattering near-field probes. The laser beam was generated by a QCL (tunable 1295–1445 cm^−1^, Daylight Solutions, USA) and focused to the tip apex using a parabolic mirror. The polarization of the illuminating beam in the presented experiments was parallel to the h-BN surface (s-polarization). The near fields scattered by the silicon tip were collected with a parabolic mirror and recorded simultaneously with the sample topography. Background contributions were suppressed by vertical tip oscillation at frequency *Ω* ≈ 300 kHz (tapping-mode AFM) and by subsequent higher harmonic demodulation of the detector signal at 4*Ω*. The modulation amplitude of the tip was around 100 nm. The amplitude and phase of the near-field components were measured with a pseudoheterodyne interferometric detection module. We recorded the out-of-plane near-field component, *E*_*z*_ (p-polarization).  Interference between the scattered p-polarized light from the tip and the reference beam was achieved by placing into the reference arm a polarizer that is rotated by 45º with respect to polarization of the illuminating beam.

### Band structure simulations

We used the finite difference time domain (FDTD) method to simulate the HPhP band structure shown in Fig. 3a. Periodic Bloch conditions were set up at the boundaries of the unit cell. Uniaxial perfect matched layers were imposed at surfaces of the cell parallel to the h-BN film. We use mesh element sizes ranging from 2 to 5 nm. The dielectric constant in cells at the h-BN-substrate and h-BN-superstrate interfaces is taken as that of the medium with the largest volume inside that particular cell. We have checked that transmittance spectra obtained in this manner coincide with those calculated using a finite elements method in frequency domain (COMSOL) (see Supplementary Note 3). The band structure is calculated by exciting the system with a superposition of randomly placed and oriented electric dipoles at different positions and imposing Bloch’s theorem at the boundaries of the unit cell. Then, the value of the amplitude of the electric fields for different Bloch phase factors (wave vector) and frequencies is calculated, revealing the band structure of the HA. The red curves in Fig. 3a trace the maxima in various colorplots (generated for different positions and orientations of the dipole sources).

### Isofrequency plots

For calculating the isofrequency plots (Fig. 3c) a finite element method in frequency domain was used (COMSOL). In the simulation we consider a finite-size HA of 27 × 27 holes. A vertical dipole source was placed 30 nm above the h-BN slab at the center of the HA. For each frequency, the spatial distribution of the radial electric field, *E*_*ρ*,h−BN_(*x*, *y*), in the plane of the dipole was calculated. The same calculation was done without the h-BN film, obtaining the distribution of the field above the substrate, *E*_*ρ*,sub_(*x*, *y*). Then, Fourier transform of the subtracted fields, *E*_*ρ*,h−BN_(*x*, *y*) − *E*_*ρ*,sub_(*x*, *y*), was performed, obtaining *E*_*ρ*_(*k*_*x*_, *k*_*y*_). To mimic the source with the electric field fulfilling the Bloch’s theorem, the following summation over different Brillouin Zones has been performed: $\mathop {\sum }\nolimits_{n,m} E_\rho (k_x + n \cdot G,k_y + m \cdot G)$. This procedure is equivalent to the monitoring of the field (satisfying the Bloch’s theorem) at the position (*x* = 0, *y* = 0).

### Analytical theory

For the reliable identification and detailed analysis of the excited Bloch mode we developed a simple analytical theory based on the thinness of the h-BN slab and resonance perturbation theory (for details see Supplementary Note 5). The h-BN is treated as a conductivity layer considering only in-plane dielectric permittivity, ${\it{\epsilon }}_ \bot$. We further consider that due to the resonance excitation of HPhPs in the hole array, the first-order Bloch field harmonics dominate. By considering only p-polarization and assuming that the plane of incidence is parallel to one of the translational vectors of the hole array, the latter can be approximated by a conductivity layer, periodically modulated in one direction (with the modulated conductivity profile having the same Fourier spectrum as the hole array). The boundary conditions for the electromagnetic field at the h-BN periodically modulated conductivity layer results in an infinite discrete set of linear equations for the spatial field harmonics. By truncating this set of equations to a diffraction order *N* = 2, the system of equations can be solved analytically, and the reflection and transmission coefficients take a simple analytical form. The poles of these coefficients provide the dispersion of the excited Bloch modes and their lifetimes.

### h-BN dielectric function

The perpendicular and parallel components of the permittivity tensor, ${\it{\epsilon }}_{zz} = {\it{\epsilon }}_\parallel$, ${\it{\epsilon }}_{xx} = {\it{\epsilon }}_{yy} = {\it{\epsilon }}_ \bot$ are approximated with a Drude–Lorentz permittivity^24^

$${\it{\epsilon }}_a\left( \omega \right) = {\it{\epsilon }}_{a,\infty }\left( {1 + \frac{{\left( {\omega _{{\mathrm{LO}}}^a} \right)^2 - \left( {\omega _{{\mathrm{TO}}}^a} \right)^2}}{{\left( {\omega _{{\mathrm{TO}}}^a} \right)^2 - \omega ^2 - i\omega \gamma ^a}}} \right).$$

Where $a = \parallel \,or\, \bot$, *ω*_LO_ and *ω*_TO_ refers to the transversal (TO) and longitudinal (LO) phonon frequencies, *γ* denotes the damping constant and ${\it{\epsilon }}_\infty$ is the high frequency permittivity. The values of the constants are: ${\it{\epsilon }}_{\parallel ,\infty } = 2.95, {\it{\epsilon }}_{ \bot ,\infty } = 4.90,\,\omega _{{\mathrm{LO}}}^\parallel = 825 \, \mathrm{cm}^{ - 1},\omega _{{\mathrm{LO}}}^ \bot = 1610 \, \mathrm{cm}^{ - 1}$, $\omega _{{\mathrm{TO}}}^\parallel = 760 \, {\mathrm{cm}}^{ - 1},\omega _{{\mathrm{TO}}}^ \bot = 1366.2 \, \mathrm{cm}^{ - 1},\gamma ^\parallel = 2 \, {\mathrm{cm}}^{ - 1},\gamma ^ \bot = 7 \, {\mathrm{cm}}^{ - 1}$. The value of $\omega _{{\mathrm{TO}}}^ \bot$ was adjusted using the position of the TO phonon dip in the transmission spectra of a flake of h-BN (See Supplementary Note 4).

## Electronic supplementary material

Supplementary Information

## Data Availability

The data that support the findings of this study are available from the corresponding author on reasonable request.
