# Supplementary material for: Deeply subwavelength phonon-polaritonic crystal made of a van der Waals material
Source: Nat Commun. 2019 Jan 3;10:42. doi: 10.1038/s41467-018-07795-6 (PMC6318287; doi:10.1038/s41467-018-07795-6)
Supplement: Supplementary file 1 — Supplementary Information [file 41467_2018_7795_MOESM1_ESM.pdf]

Supplementary Information

Deeply subwavelength phonon-polaritonic crystal made of a van der Waals material

F. J. Alfaro-Mozaz et al.

Supplementary Note 1: Electron microscope images of the h-BN hole arrays

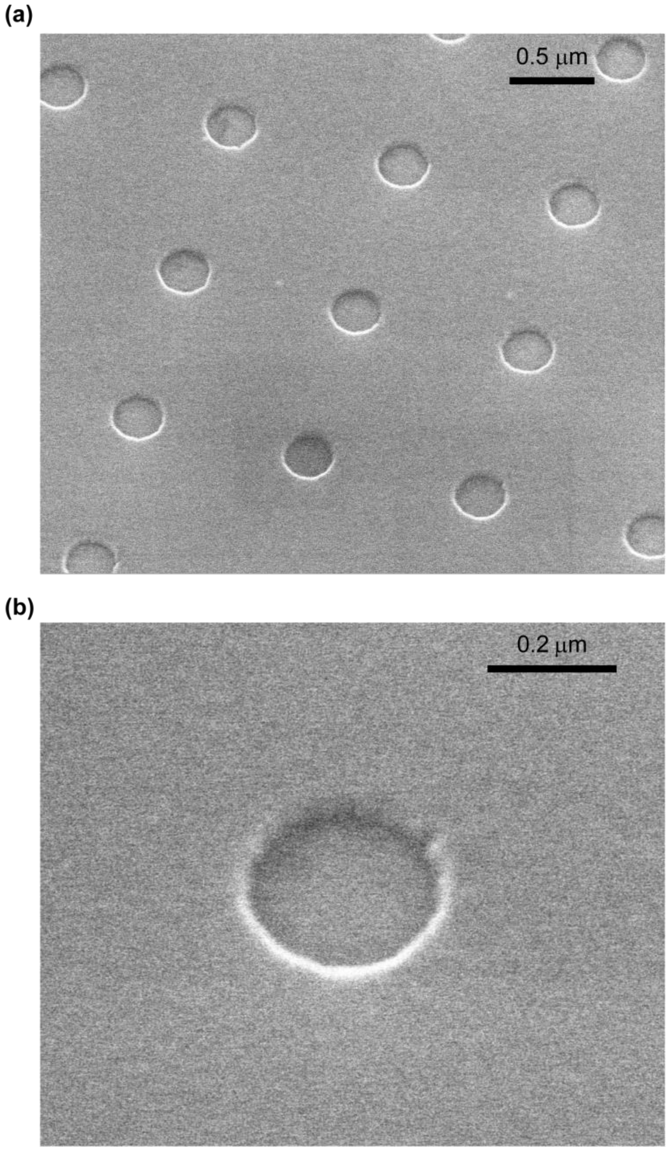

Supplementary Figure 1: Environmental scanning electron microscope (eSEM) images of a hole array with period L = 1050 nm. *The images show (a) the regularity of the fabricated array and (b) the precise lithography of a single hole.*

Supplementary Note 2: Full-wave calculations of the band structure with the excitation sources inside and outside the h-BN flake

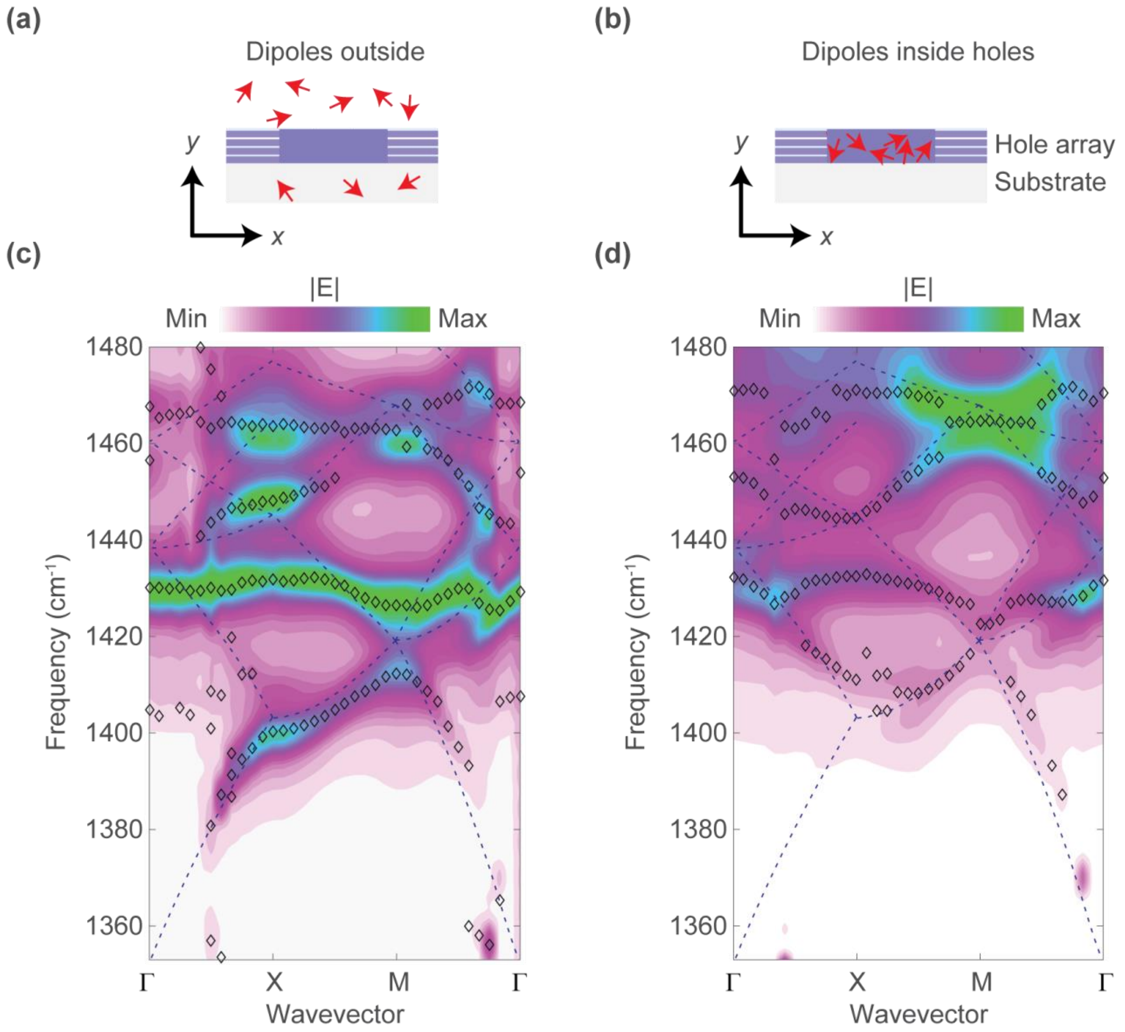

Supplementary Figure 2*: *Calculated polaritonic band structure of the hole array with L = 900 nm. *Sketch of a unit cell of the hole array illuminated with the dipole sources placed in: (a) outside of the hole array, and (b) inside the holes. The electric field "probes" (See Methods) are similarly distributed in (a) and (b). (c) and (d) shows the band structure obtained by measuring the intensity of the electric field (colorplot) when the sources are placed according to (a) and (b), respectively. The dashed lines in (c) and (d) correspond to the dispersion of the M0 polaritonic mode in the h-BN layer. The rhomb symbols in (c) and (d) represent the position of maxima in the field intensity averaged over the probes.*

The band structure (Fig. 3a) was calculated using full-wave Finite-Difference Time-Domain (FDTD) simulations (See Methods). The point dipole sources were randomly placed either outside the hole array or inside it, in the regions of the holes (but not in the

regions of the h-BN), as shown in Supplementary Figure 2a,b. In both cases the electric field was probed in randomly selected points ("probes") within the unit cell. The maxima in the electric field intensity are plotted in Supplementary Figure 2c,d (symbols), revealing the polaritonic dispersion branches. Both dipole distributions reveal the same band structure, although due to a slightly different coupling efficiency, some dispersion branches are better seen for one or another location of the dipoles. In fact, the sources inside the holes in h-BN (Supplementary Figure 2b,d) couple more efficiently with the high-energy bands, while, in contrast, the sources outside the h-BN layer (Supplementary Figure 2a,c) couple more efficiently with the low energy bands.

Supplementary Note 3: Comparison of transmission, absorption and reflection coefficients simulated by FEM and FDTD

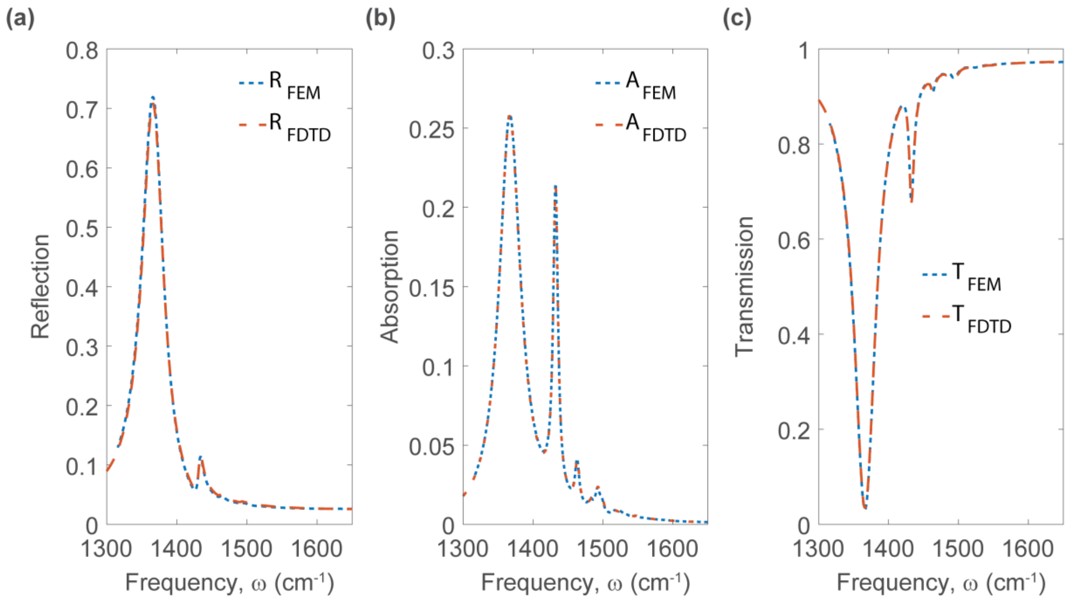

Supplementary Figure 3: Comparison of the calculated reflection, absorption and transmission through FEM and FDTD methods *(a) Reflection, R, (b) absorption, A and (c) transmission, T, through the hole array with L = 900 nm. The dashed blue and red lines correspond to a FEM (COMSOL) and FDTD method, respectively. Both calculations show an excellent agreement between them.*

In Supplementary Figure 3 we compare the transmission, absorption and reflection for a hole array with a period L = 900 nm (the results for a hole array with this period are presented in Figures 2, 3 and 4 of the main manuscript) simulated by means of both FDTD and finite elements method (FEM). The diameter of the holes is 300 nm and the thickness of the h-BN flake (placed on semi-infinite CaF_2 substrate) is 38 nm. The hole array is illuminated by a plane wave linearly polarized along one of the translation vectors of the hole array at normal incidence.

Supplementary Note 4: Comparison between experimentally measured and simulated transmission, absorption and reflection coefficients

The calculated transmission, absorption and reflection coefficients of the hole arrays are presented in Supplementary Figure 4. The reflection coefficient is about 10 % at frequencies close to the first Bragg resonance for all the measured hole arrays. On the other hand, the absorption at the first Bragg resonance frequency is comparable to that of the TO phonon (at 1366 cm^{-1}), and increases with decreasing the period, L, of the hole array. The simulated transmission spectra show an excellent agreement with the experiment in terms of spectral position, while the depth of the experimental peaks is slightly smaller than in the calculated ones. The latter discrepancy can be explained by the small aperture used in the spectrometer and the finite size of the hole array in the experiment.

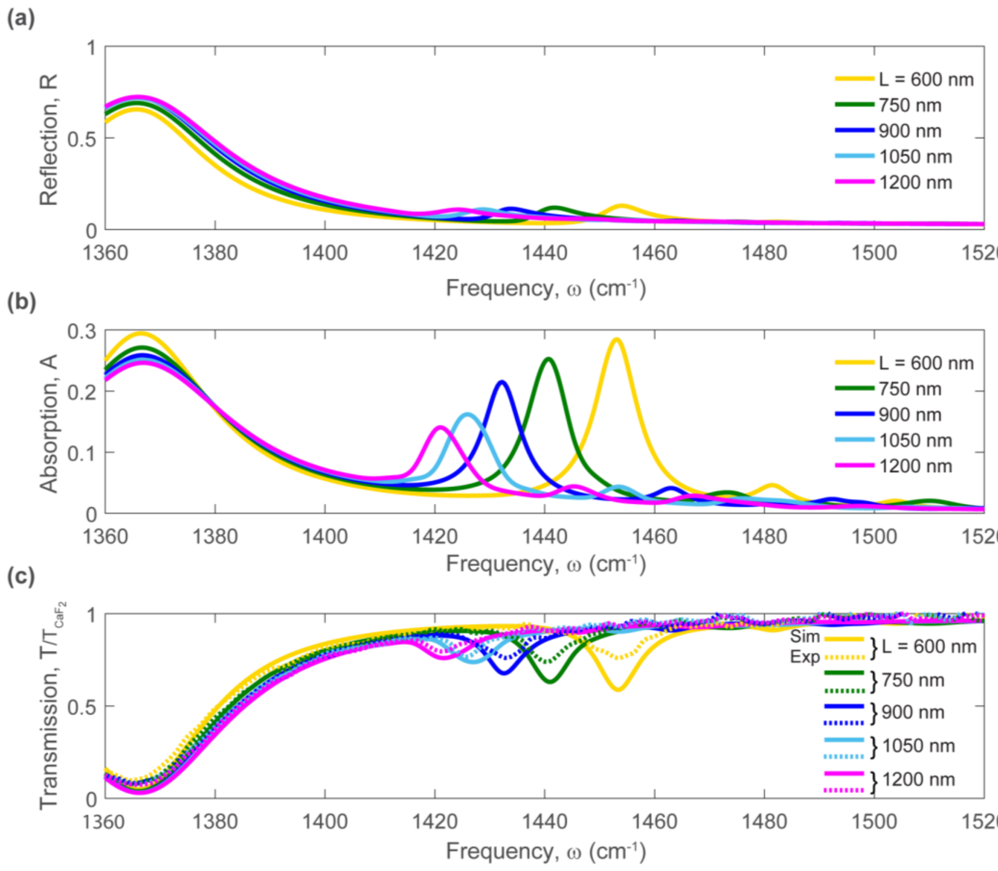

Supplementary Figure* 4: *Transmission, absorption and reflection coefficients. *(a) Reflection, (b) absorption and (c) transmission coefficients for the fabricated hole arrays shown in the main manuscript. The dashed (solid) lines correspond the experiment, (simulations).*

Supplementary Note 5: Analytical approximation

Analytical Approximation of a h-BN thin slab as a 2D conductivity layer

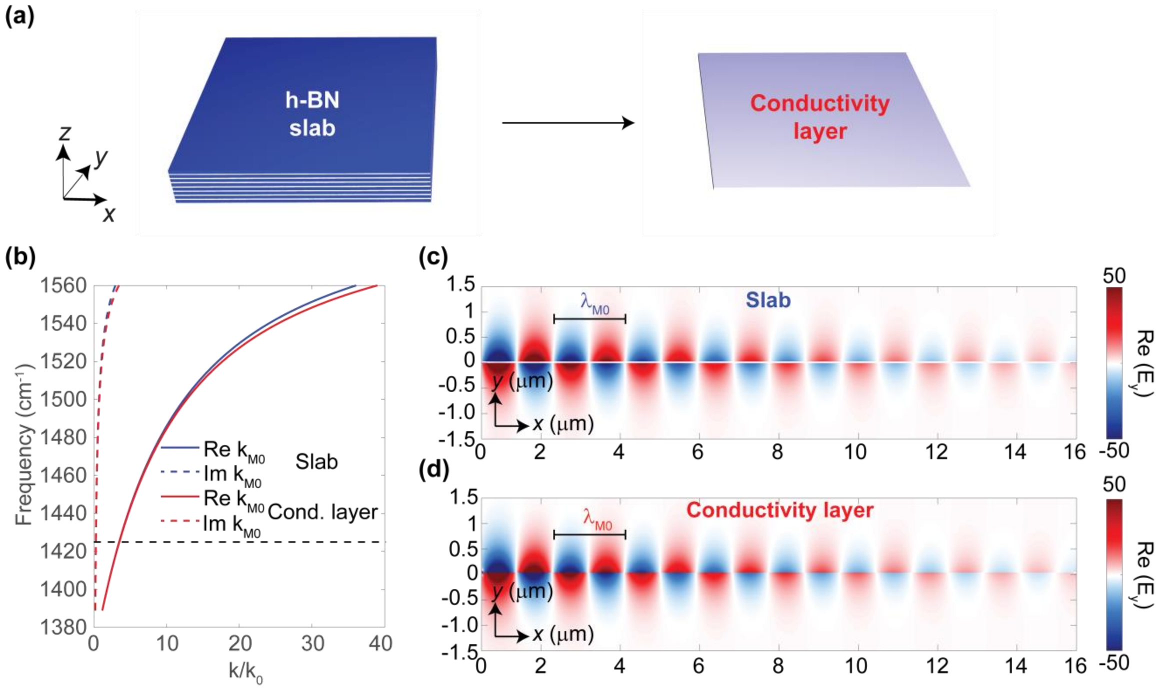

***Supplementary Figure 5**: **Dispersion and field distribution of the M0 mode in an h-BN slab and the polaritonic mode in an equivalent conductivity layer.** (a) The h-BN flake is approximated as a conductivity layer. (b) Wave-vector, k, of the M0 mode in a slab of t = 38 nm surrounded by air as a function of frequency. Solid blue line renders the real part, while the dashed blue line the imaginary part, respectively. Both real and imaginary parts of k are normalized to that of the free space light $k_0 = \frac{\omega}{c}$. (c) Simulated vertical component of the electric field, E_z (at ω=1428 cm^{-1}) of the M0 mode propagating along the x -axis (from left to right). (d) The same as in (c), but for the polaritonic mode propagating along the conductivity sheet.*

In order to get an insight into the fundamental properties of the polaritons in the hole array (e.g. lifetime or the symmetry of the modes), we model the h-BN flake as a 2D conductivity layer of zero thickness (see Supplementary Figure 5a). This approximation avoids the calculation of the fields inside the slab, and has been proven valid for in-plane isotropic 2D materials (e.g., graphene[1] and transition layer polaritons[2]) with a layer thickness, t, that is much smaller than the polariton wavelength ($t \ll \lambda_{M0}$). In the model, the effective conductivity is given by $\sigma_{\mathrm{eff}} = (ct/2i\lambda_0)\varepsilon$, where ε is the dielectric permittivity of the slab. Analogously, we model the h-BN layer by an isotropic in-plane conducting layer with zero thickness and an effective two-dimensional conductivity $\sigma_{\mathrm{eff}} = (ct/2i\lambda_0)\varepsilon_\perp$, neglecting the contribution of the in-

plane part of the dielectric permittivity of h-BN, $\varepsilon_{||}$. It is important to note that σ_{eff} scales linearly with t, thus *taking into account the effect of the slab thickness*.

We justify the validity of our model by Supplementary Figure 5, showing an excellent agreement between the dispersions (Supplementary Figure 5b) and the spatial electric field distributions (Figs. S5c, d) of the M0 mode in the h-BN slab and a polaritonic mode in the equivalent 2D conductivity layer.

Diffraction of an electromagnetic wave by the hole array in the approximation of the modulated effective conductivity layer

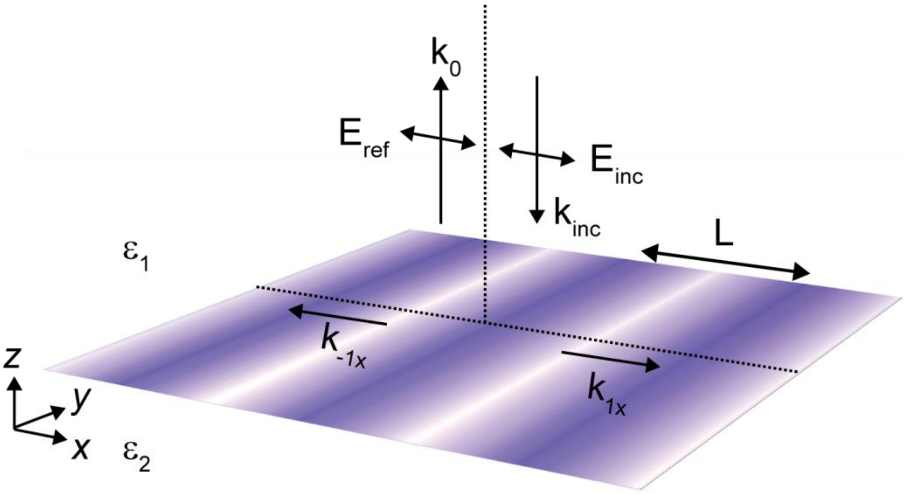

Supplementary Figure 6: Sketch of the system*. A conductivity layer is modulated in the x-direction. The linearly-polarized incident plane wave impinges at normal incidence onto the layer, exciting the first-order Bragg resonance.*

In order to analytically treat the diffraction of the electromagnetic wave by our hole array, we approximate the thin h-BN slab by a conductivity layer, following Section S5.1. In the derivations we will assume that the conductivity layer is placed between two dielectric half-spaces with the dielectric permittivities ε_1 (for $z > 0$) and ε_2 (for $z < 0$), respectively (Supplementary Figure 6). The hole array can be seen as spatially (and periodically) modulated[1] effective conductivity, and thus we will consider a general periodic structure with an arbitrary spatial Fourier spectrum.

For simplicity, we restrict ourselves to considering the incident plane wave with the in-plane electric field component parallel to one of the translation vectors of the hole array (aligned along x- and y-axes, according to the chosen system of coordinates). In this case the excited Bloch modes will be predominantly the standing waves along x- or y-directions. Neglecting the weak interaction between the standing waves excited in the perpendicular directions[3], we can approximate the hole array by two perpendicular one-dimensional (1D) periodic gratings and consider each grating independently. Without

loss of generality, let us study the 1D periodic modulation of the conductivity along the x-direction (as in Supplementary Figure 6).

The periodically modulated effective conductivity can be written as (for convenience of the equations writing, we introduce the normalized conductivity[1], α, as $\alpha = 2\pi\sigma_{\text{eff}}/c$)

$$\alpha(x) = \alpha(x+L) = \sum_n \alpha_n e^{inGx}, \qquad \text{(S5.1)}$$

with $G = \frac{2\pi}{L}$ being the reciprocal grating vector, n an integer and $\bar{\alpha}_n$ the n-th Fourier harmonic of the normalized effective conductivity. The in-plane component of the electric fields in the superstrate (labelled as "1") and substrate (labelled as "2") can be taken in the form of the Fourier-Floquet expansion:

$$\mathrm{E_{1x} = e^{ik_x x - ik_z z} + \sum_n r_n e^{ik_{nx}x + ik_{1nz}z}}, \qquad \text{(S5.2)}$$

$$\mathrm{E_{2x} = \sum_n t_n e^{ik_{nx}x - ik_{2nz}z}}, \qquad \text{(S5.3)}$$

where $k_{nx} = k_x + nG$ and $k_{1,2nz} = \sqrt{\varepsilon_{1,2}k_0^2 - k_{nx}^2}$ are the x- and z-components of the wave-vectors for the diffracted (scattered) plane waves and $\mathrm{k_0 = \omega/c}$ is the k-vector in the free space. The coefficients $\mathrm{r_n}$ and $\mathrm{t_n}$ present the amplitudes of the spatial Fourier harmonics (waves diffracted in the nth order) in the upper and lower half-spaces, respectively.

Using Maxwell's equations, $\boldsymbol{H} = (1/ik_0)\nabla \times \boldsymbol{E}$, we can find the y-component of the magnetic fields in the substrate and superstrate. Then, we match the fields at the conductivity layer ($\mathrm{z = 0}$) according to the boundary conditions

$$\mathbf{e}_z \times [\mathbf{H}_1 - \mathbf{H}_2] = 2\alpha \cdot \mathbf{e}_z \times [\mathbf{e}_z \times \mathbf{E}_1],$$

$$\mathbf{e}_z \times [\mathbf{E}_1 - \mathbf{E}_2] = 0. \qquad \text{(S5.4)}$$

Using the Fourier series for $\alpha(x)$ in Eq. (S5.1), we obtain the linear system of equations for the amplitudes $\mathrm{r_n}$ and $\mathrm{t_n}$.

$$\mathrm{e^{ik_x x} + \sum_n (r_n - t_n) e^{ik_{nx}x} = 0},$$

$$\mathrm{-Y_i e^{ik_x x} + \sum_n \left(r_n Y_{1|n} - t_n Y_{2|n}\right) e^{ik_{nx}x} = -2\sum_{n,m} \overline{\alpha}_m t_n e^{ik_{nx}x}}, \qquad \text{(S5.5)}$$

where $\mathrm{Y_i = \varepsilon_1 k_0/k_z}$ is the admittance (the inverse of the wave impedance) of the incident wave and $\mathrm{Y_{1,2|n} = \varepsilon_{1,2}k_0/k_{1,2nz}}$ are the admittances of the diffracted waves. Taking into account that Eq. (S5.5) must hold for any value of x, we have to equal the coefficients at different exponentials. Then we obtain a compact system of equations for the amplitudes $\mathrm{t_n}$:

$$\mathrm{\sum_m D_{nm} t_m = V_n},$$

$$\mathrm{D_{nm} = \delta_{nm}\left(Y_{1|n} + Y_{2|n} + 2\alpha_0\right) + 2\overline{\alpha}_{n-m}, \quad V_n = 2Y_i\delta_{n0}}, \quad \text{(S5.6)}$$

where with δ_{nm} we mean the Kronecker symbol, and $\overline{\alpha}_n$ represents the Fourier harmonic of $\alpha(x)$, given by Eq. (S5.1), but with the excluded zero harmonic ($\overline{\alpha}_0 = 0$) The amplitudes, r_n, are related to the amplitudes, t_n, as follows:

$$r_n = -\delta_{n0} + t_n. \qquad (S5.7)$$

The *infinite* linear system of equations (S5.6) can be solved numerically for any type of a periodic function $\alpha(x)$. For each type of the modulation, an appropriate *finite* number N of the diffraction orders, n, must be taken into account in order to achieve the convergence of the solution (truncation of the infinite system). In some cases, however (particularly, for periodic modulations with abrupt changes of $\alpha(x)$ as, for example, for a layer structured into ribbons), the convergence with N can be very slow and the system of equations (S5.6) becomes unpractical. On the other hand, smooth profiles of $\alpha(x)$, provide a good convergence and the number of required diffraction order is not large, so that even an analytical treatment of the system of equations is possible.

The solution of the system of equations (S5.6) can be also found analytically. Assuming small modulation amplitude, we can use the resonance perturbation theory. The main idea of the resonance perturbation theory consists in the retention in the system of equation (S5.6) all the resonant field harmonics (harmonics with high amplitudes compared to that of the incident wave), and then the minimal number of non-resonant field harmonics (originating from the lowest-order scattering of the resonant field harmonics by the diffraction grating). The number of the non-resonant field harmonics (and the contributing scattering processes) are selected with respect to the desired precision of the final solution (for more detailed description of the resonance perturbation theory in diffraction problems see Refs. 3-5). In the lowest-order approximation, the reduced system for the resonant field harmonics (whose diffraction orders we label by “r”) has the following form:

$$\sum_{r'} \widetilde{D}_{rr'} t_{r'} = \widetilde{V}_r,$$

$$\widetilde{D}_{rr'} = \delta_{rr'} b_r + 2\overline{\alpha}_{r-r'} - 4\sum_N \frac{\overline{\alpha}_{r-N}\overline{\alpha}_{N-r'}}{b_N},$$

$$\widetilde{V}_r = -4\overline{\alpha}_r \frac{Y_i}{b_0}, \qquad (S5.8)$$

where, for brevity, we have used the following notation: $b_n = Y_{1|n} + Y_{2|n} + 2\alpha_0$. In the sums of Eq. (S5.8) only non-resonant diffraction order harmonics are included (that is the resonant field harmonics with the indices r are excluded).

Normal incidence and the first-order Bragg resonance

Under normal incidence (the incident plane wave has no in-plane momentum component, i.e. $k_x = 0$), and in case of a *symmetric grating profile*, $\overline{\alpha}_m = \overline{\alpha}_{-m}$ the unknown amplitudes in the system of equations (S5.6) are symmetric with respect to the diffraction order m, i.e. $r_m = r_{-m}$ and $t_m = t_{-m}$. According to the field representation (S5.2), (S5.3), the above symmetry results in the excitation of only cos-like spatial field distributions (for the in-plane electric field) of the Bloch modes: $r_m e^{ik_{mx}x} + r_{-m}e^{-ik_{mx}x} = 2r_m \cos k_{mx}x$. This property of the inhomogeneous system of equations does not however mean that the corresponding homogeneous system of equations (with zero right-hand side) does not have solutions (eigenmodes) with other field distributions.

Let us consider the Bloch wave excited in the first-Order Bragg resonance, experimentally studied in the main manuscript. To study the Bloch (eigen-) modes, we should consider the system (S5.8), but with the right hand side. set to 0 (no incident wave). In the latter system we have only two spatial field harmonics with the amplitudes t_1, t_{-1}. It reads as

$$D t_1 + d t_{-1} = 0,$$

$$d t_1 + D t_{-1} = 0. \quad \text{(S5.9)}$$

where, for compactness, we have defined $D = \widetilde{D}_{11} = \widetilde{D}_{-1-1}$ and $d = \widetilde{D}_{1-1} = \widetilde{D}_{-11}$. The eigenfrequencies can be defined by the dispersion relation (when the determinant is set to 0)

$$D^2 - d^2 = 0. \quad \text{(S5.10).}$$

The roots of Eq. (S5.10) (which we label as S and A) can be symbolically written as $D_A = d$, and $D_S = -d$, from which the eigenfrequencies $\omega_{A,S}$ can be found. Substituting the roots back into the system (S5.9), we find two solutions for the eigenvectors:

$$t_1^S = 1, \qquad t_{-1}^S = 1$$

$$t_1^A = 1, \qquad t_{-1}^A = -1 \quad \text{(S5.11)}$$

These two solutions S and A yield the following in-plane electric field distributions on top of the grating:

$$E_{x,1}^S \sim \cos(Gx)\ , \qquad E_{x,1}^A \sim \sin(Gx). \ \text{(S5.12)}$$

For the out-of-plane electric field component (z-component), we have (consistently with the Maxwell´s equation $\nabla \boldsymbol{E} = 0$):

$$E_{z,1}^S \sim \sin(Gx), \qquad E_{z,1}^A \sim \cos(Gx). \ \text{(S5.13)}$$

Therefore, according to the correspondence between (S5.12) and (S5.13), the z-component of the mode excited under the normal incidence (the so-called *bright mode*) is distributed according to $\sin(Gx)$. The other mode, with the vertical electric field distributed according to $\cos(Gx)$ is the so-called *dark mode*.

Interestingly, depending upon the geometrical parameter of the grating (its spatial Fourier spectrum) ω_A can be either larger or smaller than ω_S, so that A is either higher- or lower-frequency mode (with respect to S). We will consider the eigenfrequencies in more details in the next section.

Dependence of the Bloch modes upon the grating (hole array) parameters

In an explicit form, the Eq. (S.5.10) can be rewritten as:

$$(\mathrm{b}-4\Gamma_1)^2-(2\overline{\alpha}_2-4\Gamma_2)^2=0, \qquad \text{(S5.14)}$$

where we have introduced the following notations:

$$b=b_1=b_{-1},$$

$$\Gamma_1=\sum_{\mathrm{N}}\frac{\overline{\alpha}_{1-\mathrm{N}}\overline{\alpha}_{\mathrm{N}-1}}{\mathrm{b_N}}, \qquad \Gamma_2=\sum_{\mathrm{N}}\frac{\overline{\alpha}_{1-\mathrm{N}}\overline{\alpha}_{\mathrm{N}+1}}{\mathrm{b_N}}, \qquad \text{(S5.15)}$$

where the summation is realized in all the diffraction orders, except 1 and -1. Let us write the solutions of Eq. (S5.14), as $\omega=\omega_0+\delta\omega$ by introducing a small deviation from the frequency of the polaritons in the unmodulated conductivity layer (with the normalized conductivity α_0), ω_0, with the dispersion relation $b(\omega_0)=0$. The latter equation (in the large momentum/short lattice period approximation, $G\gg\varepsilon_{1,2}\mathrm{k}_0$, providing under the normal incidence $\mathrm{Y}_{1,2|1}\simeq\varepsilon_{1,2}\mathrm{k}_0/\mathrm{iG}$) yields $\omega_0=-i\alpha_0\cdot\frac{2Gc}{\varepsilon_1+\varepsilon_2}$. To explicitly find $\delta\omega$ from Eq. (S5.14), we will assume that all the quantities in Eq. (S5.14) are taken at $\omega=\omega_0$, except $b(\omega)=b(\omega_0+\delta\omega)$. By expanding $b(\omega)$, we have:

$$b(\omega)\simeq\mathrm{b}(\omega_0)+\frac{\varepsilon_1+\varepsilon_2}{iGc}\delta\omega. \qquad \text{(S5.16)}$$

From Eq. (S5.14) we find the two values for $\delta\omega$:

$$\delta\omega_{A,B}=\frac{iGc}{\varepsilon_1+\varepsilon_2}[4\Gamma_1\pm(2\overline{\alpha}_2-4\Gamma_2)]. \quad \text{(S5.17)}$$

Then the expressions for the eigenfrequencies of the modes A and S become

$$\omega_A=\omega_0+\frac{4iGc}{\varepsilon_1+\varepsilon_2}\Gamma_1+\Delta\omega,$$

$$\omega_S=\omega_0+\frac{4iGc}{\varepsilon_1+\varepsilon_2}\Gamma_1-\Delta\omega. \qquad \text{(S5.18)}$$

with

$$\Delta\omega = \frac{2iGc}{\varepsilon_1+\varepsilon_2}(\overline{\alpha}_2 - 2\Gamma_2). \qquad \text{(S5.19)}$$

We see that both eigenfrequencies are shifted with respect to ω_0 by the term proportional to Γ_1 (the shift of the center of the bandgap). This term is contributed from the second-order scattering processes (via non-resonant diffraction orders) between the bare polaritons given by the resonant diffraction orders 1 and -1. The splitting, defined by $\Delta\omega$, is contributed from both second-order processes (term proportional to Γ_2) and the linear interaction between the bare polaritons (term proportional to $\overline{\alpha}_2$).

To analyze $\Delta\omega$, for simplicity, we neglect the ohmic losses, assuming that the effective conductivity (and all its Fourier harmonics) is purely imaginary. We also assume that for a weak modulation, the second-order lattice harmonic, $\overline{\alpha}_2$, exceeds the term $2\Gamma_2$ so that the linear interaction dominates over the second-order scattering. Then according to Eq. (S5.19) the sign of $\Delta\omega$ is mainly determined by the sign of the second Fourier harmonic of the grating, $\bar{\alpha}_2$, multiplied by the imaginary unit. Namely, when $\text{sign}(i\bar{\alpha}_2) < 0$, the S mode (bright mode) has a higher frequency, "ω^+", while the A mode (dark mode) has a lower frequency, "ω^-". In contrast, when $\text{sign}(i\bar{\alpha}_2) > 0$, the S mode (bright mode) has a lower frequency, "ω^-", while the A mode (dark mode) has a higher frequency, "ω^+". Taking into account that the imaginary part of the normalized effective conductivity of the unmodulated layer, α_b, is positive, the condition of $\text{sign}(i\bar{\alpha}_2) > 0$ ($\text{sign}(i\bar{\alpha}_2) < 0$) is equivalent to $\text{sign}(\bar{\alpha}_2/\alpha_b) < 0$ ($\text{sign}(\bar{\alpha}_2/\alpha_b) > 0$), respectively (neglecting the losses).

In order to apply the results of our approximate analysis directly to the case of the hole array, let us write the modulation of the normalized effective conductivity of the hole array as $\alpha(x,y) = [1 - f(x,y)] \cdot \alpha_b$, where f takes 1 (0) value inside (outside) of the holes, respectively. The Fourier transform of $f(x,y)$ in case of a rectangular array (with period L) of circular holes (with radius a) is $f_{n,m} = \frac{a}{L}\frac{1}{\sqrt{n^2+m^2}}J_1(2\pi\frac{a}{L}\sqrt{n^2+m^2})$, with J_1 being the Bessel Function of the first kind. The spectrum of the 1D grating, equivalent to the hole array can be obtained from the 2D Fourier harmonics of the normalized effective conductivity, $\bar{\alpha}_{n,m}$, by setting $\bar{\alpha}_n = \bar{\alpha}_{n,0}$. It reads:

$$\frac{\bar{\alpha}_n}{\alpha_b} = -\frac{a}{L}\frac{1}{n}J_1(2\pi\frac{a}{L}n). \qquad \text{(S5.20)}$$

When the diameter of the hole changes, the weight of the second harmonic of the Fourier decomposition of the hole array varies (see Supplementary Figure 7). According to our approximation, in the range of $a/L < 0.3$ (where $\text{sign}(\bar{\alpha}_2/\alpha_b) < 0$), the modes S and A are expected to have low (ω^-) and high (ω^+) frequencies, respectively (blue region in Supplementary Figure 7). In contrast, for $a/L > 0.3$ ($\text{sign}(\bar{\alpha}_2/\alpha_b) > 0$), the modes S and A should have high (ω^+) and low (ω^-) frequencies, respectively (green region in Supplementary Figure 7).

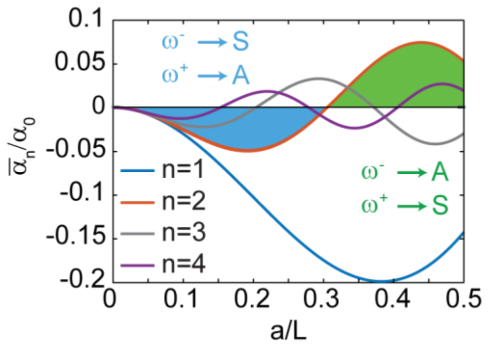

Supplementary Figure 7: Amplitude of the first four Fourier harmonics of the rectangular hole arrays as a function of the ratio between radius and array period, a/L. *The sign of the second harmonic approximately dictates the position of the eigenfrequencies relative to each other.*

We verify the results of our analytical analysis by performing the full-wave simulations (COMSOL) for the two hole arrays (placed on CaF_2 substrate) with radii of holes $a = 300$, and 150 nm (Figs. S8a,b), and period $L = 900$ nm. The parameters of the arrays have been chosen in such a way that the arrays with the smallest and largest a correspond to the blue and green areas in Supplementary Figure 7. The simulated absorption, as a function of frequency, ω, and angle of incidence, θ, is shown in Supplementary Figure 8b,c (in Section 5.5 we also show the comparison between the full-wave simulations and the numeric solution of the approximate system (S5.6)). For the hole array with $a = 300$ nm ($a/L = 0.33$) we have $\mathrm{sign}(\bar{\alpha}_2/\alpha_b) > 0$ (blue region in Supplementary Figure 7) and as predicted above, the bright mode S has a higher frequency than the dark mode A. In contrast, for the hole array with $a = 150$ nm ($a/L = 0.167$) we have $\mathrm{sign}(\bar{\alpha}_2/\alpha_b) < 0$ (blue region in Supplementary Figure 7) and the bright mode S has a lower frequency than the dark mode A. As seen in the colorplots of Fig S8b and c, for the oblique incidence the incident wave breaks the symmetry and both A and S modes are excited (this is particularly seen for angles $\theta > 20^o$). Thus, the numeric simulations corroborate our analytical results, revealing the key role of the second harmonic of the hole array in the relative frequency position of the Bloch modes in the first-order Bragg resonance.

The results of our analysis are consistent with previous studies for one- and two-dimensional metallic diffraction gratings and hole arrays in metal films (although these studies were limited to the periods comparable to the free-space wavelength in both cases)[6-8].

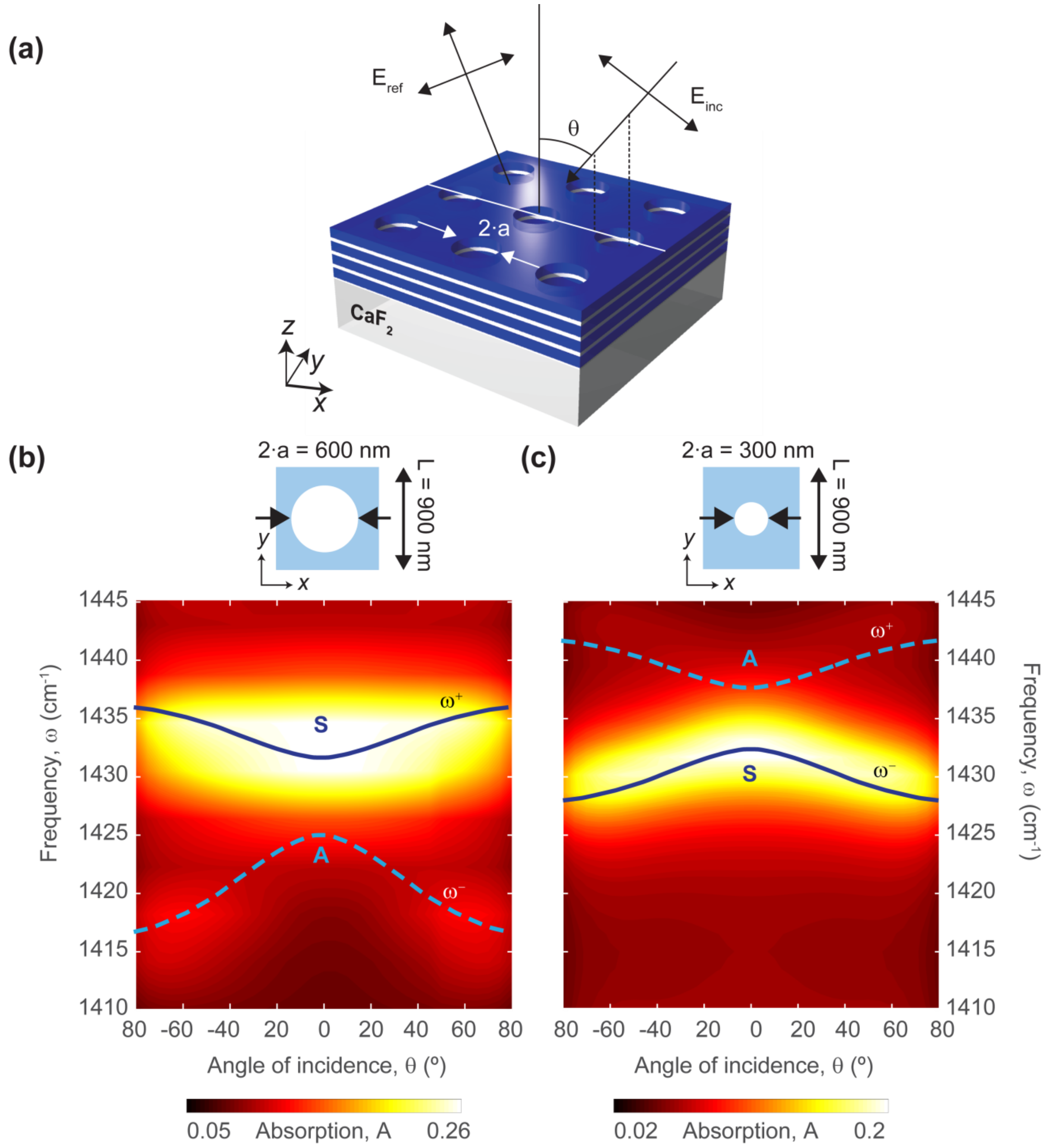

Supplementary Figure 8: Calculated normalized absorption as a function of angle for two different radii of the holes. *(a) Sketch of the hole array (L = 900 nm) on a* CaF_2 *substrate. A p-polarized wave impinges the hole arrays with an angle* θ *with respect to the normal. (b,c) Calculated absorption in the hole arrays (colorplot) as a function of* θ *and* ω*.The sketches show the relative size of the hole with respect to the period. The solid and dashed lines are guides to the eye, highlighting the position of the bright (S) and dark (A) modes, respectively.*

Analytical approximation versus full-wave simulations.

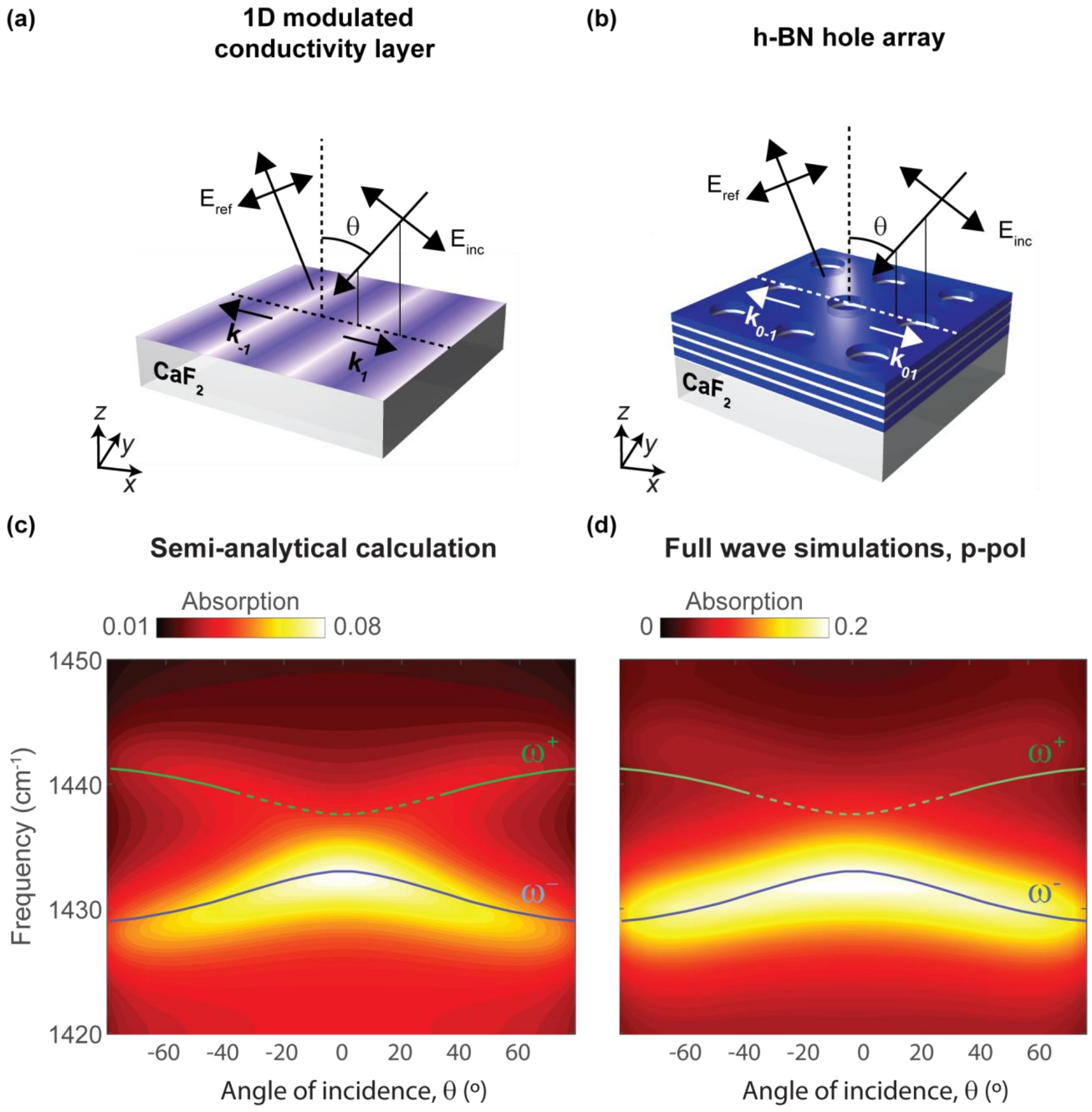

Supplementary Figure 9: Absorption of a p-polarized incident wave by the BN hole array, as a function of the angle of incidence and frequency. *(a) Schematics of the 1D modulated conductivity layer over a* CaF_2 *substrate. The incident wave excites the Bloch mode formed by the field harmonics with the k-vectors* k_{-1} *and* k_1 *(first-order diffracted waves), giving rise to the first-order Bragg resonance. (b) Schematics of the hole array. The excited Bloch mode is formed by the field harmonics with the k-vectors* k_{0-1} *and* k_{01} *. The period of both the hole array and the 1D periodic lattice is L = 900 nm. The plane of incidence (marked by white solid line) is parallel to the lattice vector in (a) and the rows of holes in (b), respectively. (c) and (d) show the absorption as the function of* θ *and* ω *(the colorplots), with the help of the system of equations (S5.6) and full-wave simulations, respectively. The blue and green curves represent the dispersion of the modes S and A, respectively.*

To further corroborate the validity of our analytical approximation developed in Section S5.2, we compare the absorption in the hole array calculated with the help of full-wave simulations (COMSOL) and the linear system of equations (S5.6). For the solution of the system of equations S5.6 we use the expression for the amplitudes of the Fourier harmonics of the periodically-modulated normalized effective conductivity, $\alpha(x)$, given by Eq.(S5.19). As before, we assume that the hole array is placed on the CaF_2 substrate. The results of the full-wave simulations and calculations according to Eq.(S5.6) are shown in Supplementary Figure 9, demonstrating an *excellent agreement between the two approaches* (compare the colorplots in Supplementary Figure 9c and and 9d). Apart from the absorption, in Fig.S9c,d we also show the dispersion branches of both modes A and S (green and blue curves, respectively), analyzed in details in Sections S5.3-5.5. The mode S (lower frequency branch) perfectly matches with the bright maximum in the colorplots. The dispersion curves were obtaining by analyzing the zeros of the determinant of the system of equations (S5.6).

Lifetime of the Bloch modes excited in the first-order Bragg resonance

In this Section we find and analyze the radiative lifetime of the Bragg mode S, excited in the first-order Bragg resonance under normal incidence. In our analysis, we will take into account only the first-order Fourier harmonic of the modulated conductivity. We assume that this harmonic plays the dominating role in both the excitation of the first-order polaritonic Bloch modes and their radiative coupling. Thus, we assume that the conductivity profile is given by the following relation:

$$\alpha(x) = \alpha_B + \Delta\alpha\cos(Gx), \quad \text{(S5.21)}$$

so that only the first-order Fourier harmonic $\overline{\alpha}_1 = \Delta\alpha/2$ is different from zero. In Eq. (S5.21) $\Delta\alpha$ is the modulation amplitude. For the conductivity profile given by Eq. (S5.21), the dispersion relation of the S mode (given by Eq.(S5.14)) simplifies to:

$$\mathrm{b}_1 - \Delta\alpha^2\left(\frac{2}{\mathrm{b}_0} + \frac{1}{\mathrm{b}_2}\right) = 0. \quad \text{(S5.22)}$$

The imaginary part of the complex root of Eq. (S5.21), $\mathrm{Im}(\omega_S)$, represents the inverse lifetime, in which both the *radiative* and *ohmic* contributions can be extracted.

Aiming on the qualitative description of the results, we will assume that the dielectric permittivity of the substrate is 1 (so that the conductivity layer stays in the *symmetric vacuum surrounding*). This assumption simplifies the writing of equations, but does not affect the generality of the lifetime dependence upon the lattice parameters. The admittances Y_n appearing in Eq. (S5.21) can be explicitly written as $\mathrm{Y}_{1,2|0} = 1;\ \mathrm{Y}_{1,2|1} \simeq \frac{-i\omega}{Gc};\ \mathrm{Y}_{1,2|2} \simeq \frac{-i\omega}{2Gc}$, where we assume that the reciprocal lattice vector, G, is much higher than the wavevector of light in free space.

The dispersion relation of polaritons in the unmodulated conductivity layer reads as $b_1 = 0$. This equation, simplifies to $\omega_0 = -i\alpha_{\mathrm{B}}(\omega_0) \cdot Gc$ (see Section S5.4) and determines the frequency of the bare polaritons in the unmodulated layer, ω_0.

Let us find the explicit expression for the effective normalized conductivity. We take the perpendicular dielectric permittivity of h-BN according to Ref [9]:

$$\varepsilon_{\perp} = \varepsilon_{\infty} + \varepsilon_{\infty}\left(\frac{\omega_{LO}^2-\omega_{TO}^2}{\omega_{TO}^2-\omega^2-i\gamma\omega}\right). \qquad \text{(S5.23)}$$

Then, following the procedure developed in Section S5.1, the effective normalized conductivity of the layer can be written as

$$\alpha_B(\omega) = A\frac{i\omega}{\omega^2-\omega_{TO}^2+i\gamma\omega}, \qquad \text{(S5.24)}$$

where $A = \frac{t}{2c}(\omega_{LO}^2 - \omega_{TO}^2)\varepsilon_{\infty}$, with d being the h-BN thickness. In Eq. (S5.23) we neglect the frequency-independent term, since the Lorentzian contribution presents a large negative value in the region of interest. Assuming that $\gamma \ll \omega$, the real part of the conductivity (responsible for the ohmic losses) is small compared to the imaginary part, $\mathrm{Re}(\alpha) \ll |\mathrm{Im}(\alpha)|$. Additionally, we assume the modulation amplitude is small, $|\Delta\alpha| < \alpha_B$. Therefore, we can consider the imaginary part of the frequency as a perturbation to $\omega = \omega_0$. Let us take into account both the perturbation due to the ohmic losses and lattice as $\delta\omega$, so that the frequency of the mode S can be written as $\omega_S = \omega_0 - i\delta\omega$. Then we can expand Eq. (S5.22) into the Taylor series (considering both $\delta\omega$, γ and $\Delta\alpha$ to be the small parameters):

$$-2\frac{i(\omega_0-i\delta\omega)}{Gc} + 2\alpha|_{\substack{\omega=\omega_0\\ \gamma=0}} - 2\frac{\partial\alpha}{\partial\omega}\Big|_{\substack{\omega=\omega_0\\ \gamma=0}} \mathrm{i}\delta\omega + 2\frac{\partial\alpha}{\partial\gamma}\Big|_{\substack{\omega=\omega_0\\ \gamma=0}}\gamma - \Delta\alpha^2\left(1+\frac{iGc}{2\omega_0}\right) = 0, \qquad \text{(S5.25)}$$

where due to small value of α, we have replaced b_0 and b_2 by $2\mathrm{Y}_{1|0}$ and $2\mathrm{Y}_{1|2}$, respectively. Taking into account the dispersion relation for the bare polaritons in the unmodulated slab, the correction to the frequency can be explicitly found from Eq. (S5.25):

$$\delta\omega = -\frac{\Delta\alpha^2\left(1+\frac{iGc}{2\omega_0}\right)-2\frac{\partial\alpha}{\partial\gamma}\Big|_{\substack{\omega=\omega_0\\ \gamma=0}}\gamma}{\frac{2}{Gc}+2i\frac{\partial\alpha}{\partial\omega}\Big|_{\substack{\omega=\omega_0\\ \gamma=0}}}. \qquad \text{(S5.26)}$$

This equation can be written in the form of the sum of the two terms: $\delta\omega_0$ (independent upon $\Delta\alpha$) and $\delta\omega_{rad}$ (independent upon $\Delta\alpha$), presenting the inverse ohmic and radiative lifetimes, τ_0 and τ_{rad}, respectively:

$$\delta\omega = \delta\omega_0 + \delta\omega_{rad} = \tau_0^{-1} + \tau_{rad}^{-1}. \qquad \text{(S5.27)}$$

Taking the derivatives $\frac{\partial\alpha}{\partial\omega}$ and $\frac{\partial\alpha}{\partial\gamma}$, the correction related to the ohmic losses becomes

$$\delta\omega_0 = \tau_0^{-1} = \frac{\gamma}{2}. \qquad \text{(S5.28)}$$

The polariton lifetime due to the intrinsic losses in a thin h-BN slab is thus *independent upon the film thickness*.

The radiative correction to the resonant frequency is given by (we use the explicit expression for α_B from Eq. (S5.23) at $\gamma = 0$ and $\omega = \omega_0$)

$$\delta\omega_{rad} = -\frac{1}{4A}\cdot\Delta\alpha^2\cdot\frac{\left(\omega_0^2-\omega_T^2\right)^2}{\omega_0^2}. \qquad \text{(S5.29)}$$

In this expression we do not include the term proportional to $\frac{iGc}{2\omega_0}$ (coming from the denominator of Eq. (S5.26)), since the latter contributes to the frequency shift (contributes to the real part of frequency). As we see, the radiative correction is proportional to the squared modulation amplitude and originate from the forward and back-scattering of the 1st diffraction orders (mainly composing the Bloch polariton) via the 0th order field harmonic.

According to Eqs. (S5.1), (S5.20) and (S5.21), the amplitude of the first Fourier harmonic of the hole array is $\alpha_1 = \frac{\Delta\alpha}{2} = -\alpha_B\frac{a}{L}J_1(2\pi\frac{a}{L})$. Substituting $\Delta\alpha$ into Eq. (S5.29) (we use α_B given by Eq.(S5.24) at $\gamma = 0$ and $\omega = \omega_0$), we have the following explicit expression for the radiative lifetime, as a function of the parameters of the hole array:

$$\delta\omega_{rad} = \tau_{rad}^{-1} = A\cdot\left(\frac{a}{L}\right)^2\cdot J_1^2\left(2\pi\frac{a}{L}\right). \qquad \text{(S5.30)}$$

Notice that in contrast to the ohmic lifetime, the radiation lifetime is thickness-dependent (via the factor $A \propto t$, introduced in Eq. (S5.24)).

To corroborate our analytical analysis, we analyze the total lifetime of the polaritons, τ_{tot}, in the hole array with the help of the full-wave simulations and experimental data. We extract the lifetime from both the simulated and experimental transmission spectra of several hole arrays (with different periods L) by fitting them to Lorentzian profiles. The extracted experimental values for τ_{tot} (triangles) are plotted in Supplementary Figure 10a as a function of period, L, finding a very good agreement with the simulations (red curve). Both red curve and the triangular symbols show a clear dependence upon L, deviating from the constant value of the ohmic lifetime, τ_0, (calculated according to Eq. S5.28), traced by the black solid curve. This deviation is due to the finite radiative lifetime, which can be extracted from the total lifetime as $\tau_{rad}^{-1} = \tau_{tot}^{-1} - \tau_0^{-1}$. In the shown interval of L, the radiative lifetime, τ_{rad}, (Supplementary Figure 10a, green curve) is larger than τ_0 up to one order of magnitude

(10 ps vs 1 ps for L = 1200 nm), indicating that the polaritons require significantly more time to couple to the free-space radiation than to be dissipated into the heat.

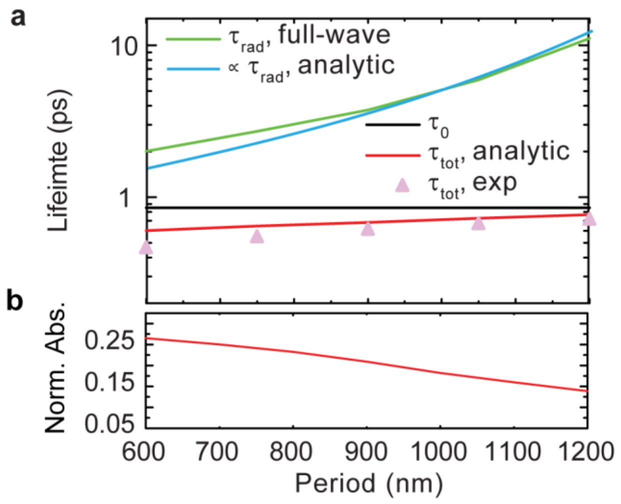

Supplementary Figure 10: Radiative and ohmic lifetimes of the Bloch mode as a function of the period of the hole array. *(a) Total, radiative and ohmic lifetime of the polaritonic Bloch mode excited in the hole array as a function of its period. (b) Absorption as a function of period.*

The result of Eq. (S5.30) (corrected by a constant factor) is shown in Supplementary Figure 10a by the blue continuous curve. The tendency provided by Eq. (S5.30) finds a good agreement with τ_{rad} extracted from the full-wave simulations (Supplementary Figure 10a, green curve). This agreement indicates that the mechanism of the radiative losses of the polaritonic Bloch mode (excited in the first-order Bragg resonance) is consistent with the scattering of the Bloch mode via the first-order harmonic of the hole array into the diffracted wave of 0th order.

As τ_{rad} approaches τ_0 (with L decrease), the polariton-induced absorption of light by the hole array increases (Supplementary Figure 10b). In fact, the optimal absorption is expected under the general condition of the equality between the radiative and ohmic losses, $\tau_{rad} = \tau_0$, which can be achieved by optimizing the aspect ratio a/L, thickness of the h-BN slab and the symmetry of the array (e.g. considering the hole arrays of triangular or hexagonal symmetries).

Supplementary Note 6: Experimental study of the transmission resonances upon the incident angle and polarization of light

The FTIR setup (Bruker Hyperion 2000, see 'Methods'), that we use for angle-dependent spectroscopy experiments, consists in two identical Swartzschild lenses. In transmission mode, the first lens focalizes the polarized light onto the sample (upper part of Supplementary Figure 11a, lens 1), while the second lens collects the transmitted light (lower part of Supplementary Figure 11a, lens 2). In this configuration, the

incident beam spans a range of plane waves with the angles of incidence, θ, ranging from $\theta_1 = 9.8°$ to $\theta_1 = 23.8°$. The sample with the hole array with period $L = 900$ nm is rotated in the xy -plane (see Supplementary Figure 11a) by an angle ϕ ranging from $-40°$ to $40°$. Although ϕ does not directly represents the angle of incidence, its change is equivalent to the change of the incident angle, θ, in the ample range between $\theta = -63.8°$ and $\theta = 63.8°$. Therefore, by showing the independence of the Bragg resonance upon ϕ, we prove that this resonance is independent upon θ.

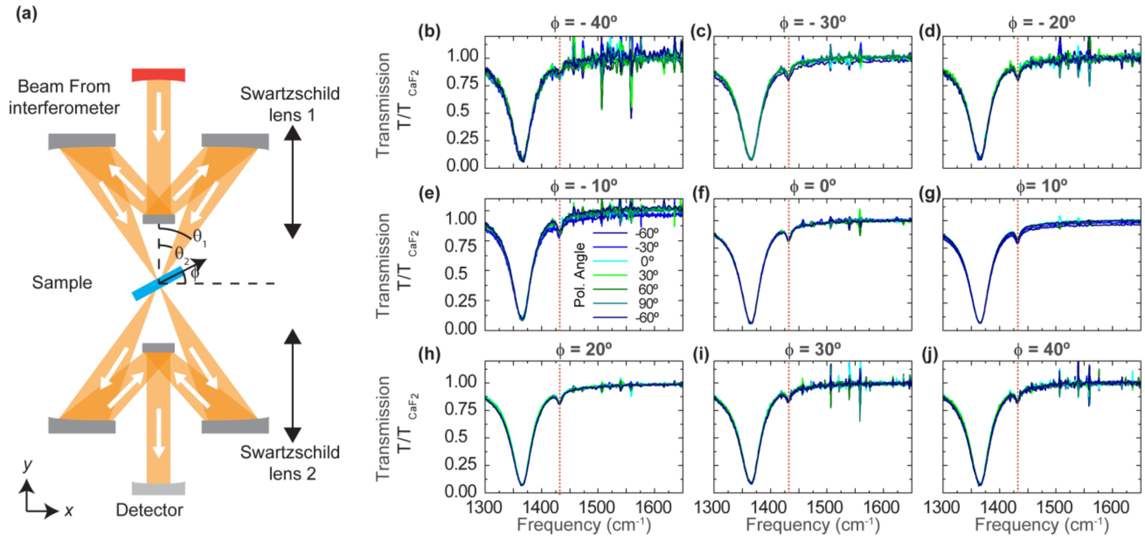

Supplementary Figure 11: Experimental infrared spectra for the hole array as a function of the stage rotation and polarization of the incident light. *(a) Schematics of the setup. The polarized beam from the interferometer is focused through a Swartzchild lens 1 providing the waves with the incident angles ranging from* $\theta_1 = 9.8°$ *to* $\theta_2 = 23.8°$*. The beam is focused onto the h-BN hole array. The transmitted light is then refocused by the Swartzchild lens 2 that sends the beam to the detector. (b-j) Experimental infrared spectra of the hole array (with L = 900 nm) for different polarization directions and tilting of the sample.*

To prove the independence of the transmission Bragg resonance upon ϕ, we have performed the spectral measurements at many rotation angles, ϕ, in the interval (mentioned above) allowed by the setup. For each rotation angle, ϕ, the measurements have been done for several polarization angles (the polarization angle is defined as the rotation of the in-plane electric field in the incident beam with respect to one of the translation vectors of the hole array). The results of the measurements (summarized in Fig. 4 of the main manuscript) for several selected values of ϕ are shown in Supplementary Figure 11b-j. The transmission spectra are normalized to that of CaF_2 substrate at the same rotation angle and polarization. The transmission resonant dip remains at the constant spectral position ($\omega = 1432$ cm^{-1}, marked by the vertical red line), maintaining a similar depth for all values of ϕ. The polarization angle (its zero value corresponds to p-polarized light) changes, the transmitted spectra remain the same, thus confirming that the measured resonance is both *angle- and polarization-independent*. Note that the noise in the spectrum increases with increasing ϕ . The noise increase can be explained by both the partial blocking of the beam by the stage and the smaller effective area of the aperture at the higher angles. The origin of the noise comes

from the imperfect cancellation of the atmospheric water lines due to small changes in the chamber between the recording of the reference and hole array interferograms.

Supplementary Note 7: Propagation length of the M0 mode in the unpatterned h-BN slab

Comparison of the analytical propagation length with the array period

To corroborate the identification of the resonant transmission peaks as Bragg resonances in the hole array, we plot the propagation length of the M0 mode (solid blue line), obtained from the quasistatic solution of the M0 mode dispersion relation in the unpatterned h-BN slab. The propagation length is calculated as $l_{M0} = \frac{1}{k''_{M0}}$. In Supplementary Figure 12 We compare l_{M0} with the period of each hole array, L, at the frequencies of the first-order Bragg resonance (grey bars). We find that l_{M0} is much higher than L, thus confirming that the M0 mode can indeed build up the Bragg resonance.

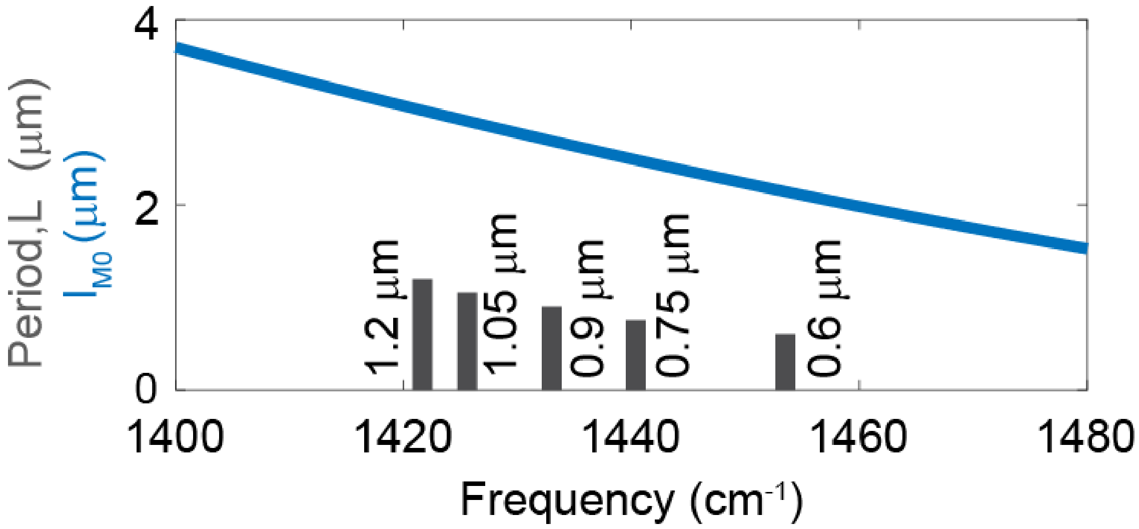

Supplementary Figure 12: Propagation length of the M0 mode as a function of frequency compared with the distance between holes at the resonances. *The solid blue line represents the propagation length of the M0 mode. The gray bars placed at the frequencies of the first-order Bragg resonance indicate the period of the hole arrays.*

Real-space imaging of the M0 mode in the unpatterned slab

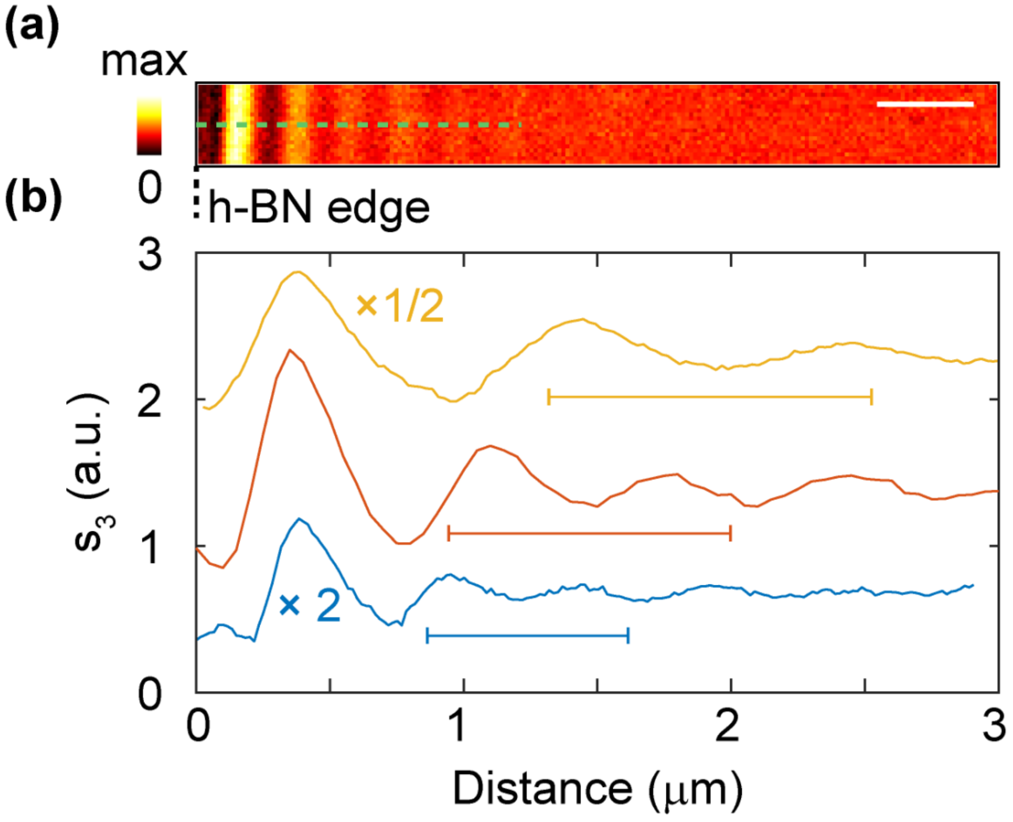

Supplementary Figure 13: Real-space s-SNOM polariton interferometry imaging of the M0 mode in the unpatterned h-BN slab. *In the polariton interferometry experiment[10-12] a metallized tip concentrates the incident light of the laser at its apex, thus acting as a launcher of the M0 mode. This mode emanates radially from the tip towards the edge of the unpatterned h-BN flake. Upon reflection, the M0 mode propagates backward and interferes with the emanating mode. The tip scatters the interfering field, providing a detectable signal in the far field. The tip raster-scans the sample and the scattered signal is recorded as a function of the tip position, providing the near-field images.* ***(a)*** *The real-space image of the unpatterned region (near the edge) of the h-BN flakes with the hole-arrays. The h-BN edge is shown by the vertical black dashed line. The frequency of the illuminating laser is 1428 cm^{-1} and the scale bar is 1.2 μm, corresponding to the longest period of the hole arrays.* ***(b)*** *Near-field line profile in the direction perpendicular to the h-BN flake edge (the direction is shown by the green dashed horizontal line in (a)) for three different frequencies: 1418 cm^{-1}, 1428 cm^{-1} and 1445 cm^{-1} from top to bottom, respectively. The profiles have been vertically shifted to improve the representation. To reduce the noise, the profiles have been averaged over several pixels. The horizontal scale bars are 1200 nm, 1050, nm and 750 nm, (from top to bottom, respectively). They represent the periods of the arrays with frequencies of the Bragg resonances close to the frequency of the illuminating laser.*

Supplementary References

1. Nikitin, A.Y. Graphene Plasmonics. in *World Scientific Handbook of Metamaterials and Plasmonics* Vol. 4: Recent Progress in the Field of Nanoplasmonics (ed. Aizpurua, J.) (World Scientific, 2017).
2. Agranovich, V.M. & Mills, D.L. *Surface Polaritons: Electromagnetic Waves at Surfaces and Interfaces*, (1982).
3. Kats, A.V., Nesterov, M.L. & Nikitin, A.Y. Excitation of surface plasmon-polaritons in metal films with double periodic modulation: Anomalous optical effects. *Physical Review B* **76**, 045413 (2007).
4. Kats, A.V. & Nikitin, A.Y. Analytical treatment of anomalous transparency of a modulated metal film due to surface plasmon-polariton excitation. *Physical Review B* **70**, 235412 (2004).
5. Slipchenko, T.M., Nesterov, M.L., Martin-Moreno, L. & Nikitin, A.Y. Analytical solution for the diffraction of an electromagnetic wave by a graphene grating. *Journal of Optics* **15**, 114008 (2013).
6. Barnes, W.L., Preist, T.W., Kitson, S.C. & Sambles, J.R. Physical origin of photonic energy gaps in the propagation of surface plasmons on gratings. *Physical Review B* **54**, 6227-6244 (1996).
7. Yamamoto, N. & Saito, H. Size dependence of band structures in a two-dimensional plasmonic crystal with a square lattice. *Optics Express* **22**, 29761-29777 (2014).
8. Saito, H. & Yamamoto, N. Size dependence of bandgaps in a two-dimensional plasmonic crystal with a hexagonal lattice. *Optics Express* **23**, 2524-2540 (2015).
9. Caldwell, J.D. et al. Sub-diffractional volume-confined polaritons in the natural hyperbolic material hexagonal boron nitride. *Nat Commun* **5**(2014).
10. Fei, Z. et al. Gate-tuning of graphene plasmons revealed by infrared nano-imaging. *Nature* **487**, 82-85 (2012).
11. Chen, J. et al. Optical nano-imaging of gate-tunable graphene plasmons. *Nature* **487**, 77-81 (2012).
12. Nikitin, A.Y. et al. Real-space mapping of tailored sheet and edge plasmons in graphene nanoresonators. *Nat Photon* **10**, 239-243 (2016).
